# Thermoresponsive and Injectable Hydrogel for Tissue Agnostic Regeneration

**DOI:** 10.1002/adhm.202201714

**Published:** 2022-10-17

**Authors:** Dax Calder, Ali Fathi, Farshad Oveissi, Simin Maleknia, Terence Abrams, Yiwei Wang, Joanneke Maitz, Kevin Hung‐Yueh Tsai, Peter Maitz, Wojtek Chrzanowski, Ivan Canoy, Vivek Ashoka Menon, Kenneth Lee, Benjamin J. Ahern, Natasha E. Lean, Dina M. Silva, Paul M. Young, Daniela Traini, Hui Xin Ong, Rasoul Seyed Mahmoud, Hossein Montazerian, Ali Khademhosseini, Fariba Dehghani

**Affiliations:** ^1^ School of Chemical and Biomolecular Engineering The University of Sydney Sydney NSW 2006 Australia; ^2^ Faculty of Medicine and Health Nano Institute The University of Sydney Sydney NSW 2006 Australia; ^3^ Faculty of Health and Medical Sciences School of Biomedical Sciences University of Western Australia Perth WA 6009 Australia; ^4^ Tetratherix Sydney NSW 2015 Australia; ^5^ Burns and Reconstructive Surgery Research Group ANZAC Research Institute Concord NSW 2139 Australia; ^6^ Anatomical Pathology NSW Health Pathology Concord Repatriation General Hospital Concord NSW 2139 Australia; ^7^ School of Medicine University of Sydney Sydney NSW 2006 Australia; ^8^ School of Veterinary Science The University of Queensland Brisbane QLD 4072 Australia; ^9^ Macquarie Medical School Faculty of Medicine and Health Macquarie University & Woolcock Institute of Medical Research The University of Sydney Glebe NSW 2037 Australia; ^10^ Ab Initio Pharma Camperdown NSW 2050 Australia; ^11^ Terasaki Institute for Biomedical Innovation Los Angeles CA 90024 USA; ^12^ Department of Bioengineering University of California Los Angeles CA 90095 USA; ^13^ California NanoSystems Institute (CNSI) University of California Los Angeles CA 90095 USA

**Keywords:** injectable hydrogel, platform technology, regenerative medicine

## Abstract

Injectable hydrogels can support the body's innate healing capability by providing a temporary matrix for host cell ingrowth and neovascularization. The clinical adoption of current injectable systems remains low due to their cumbersome preparation requirements, device malfunction, product dislodgment during administration, and uncontrolled biological responses at the treatment site. To address these challenges, a fully synthetic and ready‐to‐use injectable biomaterial is engineered that forms an adhesive hydrogel that remains at the administration site regardless of defect anatomy. The product elicits a negligible local inflammatory response and fully resorbs into nontoxic components with minimal impact on internal organs. Preclinical animal studies confirm that the engineered hydrogel upregulates the regeneration of both soft and hard tissues by providing a temporary matrix to support host cell ingrowth and neovascularization. In a pilot clinical trial, the engineered hydrogel is successfully administered to a socket site post tooth extraction and forms adhesive hydrogel that stabilizes blood clot and supports soft and hard tissue regeneration. Accordingly, this injectable hydrogel exhibits high therapeutic potential and can be adopted to address multiple unmet needs in different clinical settings.

## Graphical abstract

A fully synthetic and ready‐to‐use injectable solution is engineered that forms an adhesive hydrogel in contact with body temperature. The hydrogel provides a temporary matrix for host cell ingrowth and tissue regeneration. Preclinical and clinical results show the potential of the technology to address multiple unmet needs in different clinical settings.

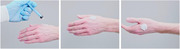

## 1 Introduction

Injectable hydrogels can have a critical role in reconstructive surgeries to support the innate regenerative capability of host tissue to expedite recovery and enhance patients' health outcomes.^[^ ^1^ ^]^ Ideally, hydrogel solutions must be easy to administer, negate the need for cumbersome preparation steps, and gelation must occur in the absence of toxic chemicals or exothermic reactions.^[^ ^2^ ^]^ Such systems are intended to adhere to the host tissue to immobilize the innate pool of progenitor cells and cytokines, naturally recruited after acute trauma. Further, the hydrogel must not induce severe foreign body reactions and fibrosis to allow cell permeation and subsequent integration with the host tissue.^[^ ^3^ ^]^ While incorporating growth factors in hydrogels to promote specific tissue healing has shown positive results in multiple preclinical settings, their clinical translation is often limited due to the requirement for supraphysiological doses, high cost,^[^ ^4^ ^]^ severe systemic side effects,^[^ ^5^, ^6^ ^]^ local impact on surrounding tissue and ectopic tissue formation.^[^ ^7^, ^8^, ^9^ ^]^ Furthermore, the specificity of these biological moieties limits their therapeutic scope and confines their use to a particular tissue type. To overcome these limitations, there has been a shift toward the application and development of several tissue agnostic injectable hydrogels devoid of any phenotypic biological activity.

Tissue agnostic injectable hydrogel systems have been formulated using several approaches, including ionic crosslinking systems from oppositely charged polymers or by combining ionizable polymer counterions from alginate,^[^ ^10^, ^11^ ^]^ polypeptides,^[^ ^12^, ^13^ ^]^ polymer–polymer chitosan with glycerophosphate,^[^ ^14^ ^]^ polyglutamate,^[^ ^15^ ^]^ or physically crosslinking systems through H‐bonding of polymer combinations of poly (*N*‐acryloyl glycinamide)/polyvinyl alcohol‐based systems^[^ ^16^ ^]^ and the mixture of two or more methylcellulose/hyaluronic acid,^[^ ^17^ ^]^ gelatin/agar,^[^ ^18^ ^]^ and starch/carboxymethyl cellulose^[^ ^18^ ^]^ or via hydrophobic interactions of amphiphilic polymers.^[^ ^19^, ^20^ ^]^ Recently, a plethora of research has emerged, focusing on diacrylate and methacrylate‐based light‐curable hydrogel systems, underpinned by their biocompatible nature and their capability to encapsulate cells while preserving high viability.^[^ ^21^, ^22^, ^23^ ^]^ However, the clinical relevance of these light‐curable hydrogels are limited to superficial tissues (1–2 mm in depth) as direct light exposure is required to induce gelation despite numerous attempts to overcome this shortcoming.^[^ ^24^, ^25^, ^26^, ^27^, ^28^ ^]^ Consequently, to develop clinically favorable polymer systems amenable to superficial and deep tissue applications, temperature‐responsive amphiphilic polymers are deemed more favorable as the gelation is triggered at physiological conditions via hydrophobic interactions.^[^ ^19^, ^29^ ^]^

Temperature responsive polymer systems have been developed using pluronic, poly(vinyl ether), poly(*N*,*N*‐diethylacrylamide‐*co*‐acrylic acid), poly(oligo(ethylene glycol) methyl ether methacrylate), poly(*N*‐isopropyl acrylamide), and chitosan.^[^ ^30^ ^]^ Poly(*N*‐isopropylacrylamide) (NIPAAm) based systems have been identified as particularly advantageous in biomedical applications due to their innate ability to copolymerize with other synthetic monomers/macromonomers via highly controllable radical polymerization techniques.^[^ ^31^ ^]^ NIPAAm is water‐soluble at neutral pH and gelates through an isothermal phase transition behavior which preserves the viability and regenerative capability of the host tissue.^[^ ^32^, ^33^, ^34^ ^]^ A fast resorption rate (in less than seven days) of NIPAAm copolymers with  2‐hydroxyethyl methacrylate lactate and acrylic acid limits the therapeutic potential of the copolymers to drug delivery applications only.^[^ ^35^ ^]^ On the other hand, the lack of degradation properties in NIPAAm copolymers with methoxy poly(ethylene glycol) methacrylate inhibits tissue ingrowth, an essential requirement for regenerative hydrogels.^[^ ^36^ ^]^ Similarly, no sign of degradation of NIPPAm copolymerized with butylacrylate hydrogels is reported despite the system's favorable gelation and adhesion characteristics.^[^ ^37^ ^]^ NIPAAm copolymers with acrylic acid‐*N*‐hydroxysuccinimide and dimethyl‐*c*‐butyrolactone fail to form 3D matrices at the administration site to support tissue healing as their low structural stability leads to the spread of the gel into a thin layer after injection. The inflammatory response and the subsequent fibrotic tissue formation around NIPAAm copolymer with acrylic acid‐macromer 2‐hydroxyethyl methacrylate‐poly(ɛ‐caprolactone) are believed to prevent ingrowth of progenitor cells and thus limit its application as a regenerative hydrogel.^[^ ^38^ ^]^ Our research group has previously developed a highly biocompatible thermoresponsive poly (NIPAAm‐*co*‐(*N*‐acryloxysuccinimide)‐*co*‐(polylactide/‐hydroxy methacrylate)‐*co*‐(oligo (ethylene glycol), denoted as PNPHO.^[^ ^39^ ^]^ Despite its promising biological properties and degradation profile, the previously developed polymer hydrogel displayed unfavorable gelation kinetics as 2–10 min were required to induce hydrogel formation under physiological conditions.^[^ ^39^ ^]^ This limits the clinical relevance of the previously developed PNPHO based hydrogel as prolonged gelation time can lead to in situ dilution and dislodgment of the hydrogel in the presence of active bleeding from the administration site. The chemical composition and physical properties of thermoresponsive based hydrogel systems are yet to be optimized to address current unmet clinical needs.

Here, we developed an injectable thermoresponsive hydrogel by optimizing the chemical composition of PNPHO and crosslinking the polymer with a synthetic peptide, namely Thymosin‐*β*4, to tune the gelation kinetics and thus the clinical usability of the resulting system. Notably, this peptide was selected as it does not elicit any cell specific phenotypic response,^[^ ^40^ ^]^ a key requirement in developing a tissue agnostic hydrogel system. The final product is denoted as TX140, where the annotation was derived from “thermoresponsive” (T) and “matrix” (X), followed by the concentration of PNPHO (mg mL^−1^) as the suffix. A comprehensive range of preclinical in vitro and in vivo animal models was used to confirm the structural stability, adhesivity, biocompatibility, and regenerative potential of TX140 at different anatomical sites. Furthermore, a human study was conducted to assess the clinical usability of TX140 and its efficacy as a platform injectable hydrogel to support the regeneration of both soft and hard tissues.

## 2 Results

### 2.1 Chemical Composition and Optimization of TX140

**Figure 1:**
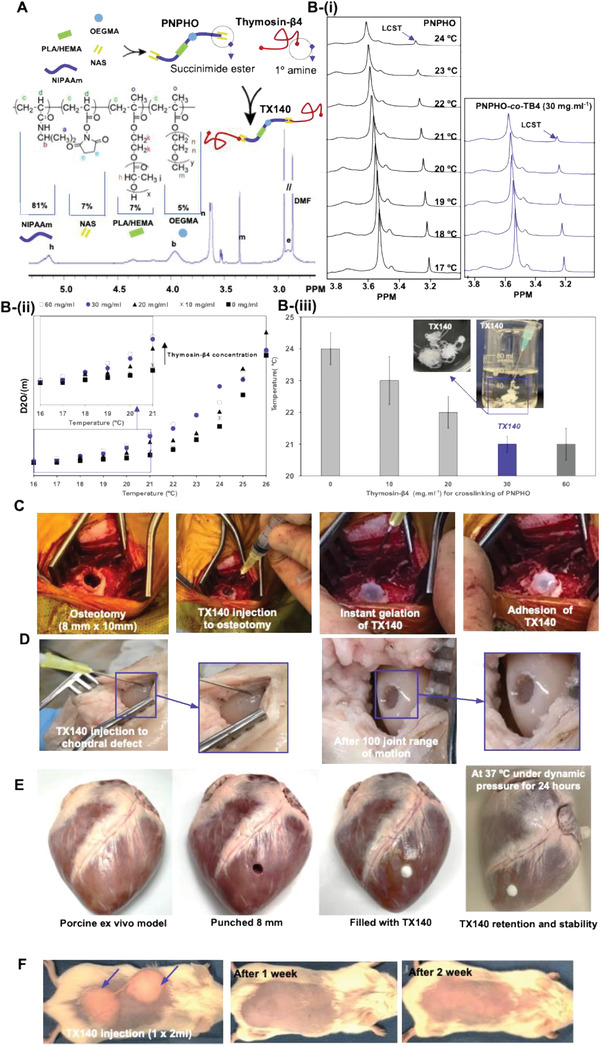
Chemical composition of TX140 and its application at different anatomical sites; A) schematic representation of PNPHO along with its chemical composition and ^1^H‐NMR spectrum of the polymer with the use of Thymosin‐*β*4 to conjugate with PNPHO. B) The region of interest, (m) peak, in ^1^H‐NMR spectra of PNPHO polymer solution i) and PNPHO‐*co*‐Thymosin‐*β*4 with 30 mg mL^−1^ at different temperatures ranging from 17 to 24 °C. D_2_O/(m) of PNPHO*‐co*‐Thymosin‐*β*4 solution at different temperature to find LCST of the solutions ii). LCST of the different PNPHO*‐co*‐Thymosin‐*β*4 solution with different Thymosin‐*β*4 concentration (iii, source data in Figure S4 (Supporting Information), and *n* = 10). C) The hydrogel is injectable and forms a matrix in physiological conditions in a live sheep osteotomy model (*n* = 6). D) Adhesion and retention of TX140 in an ex vivo bovine cadaveric subchondral defect model without the need for physical containment in a dynamic model after 100 cycles of complete joint motion (*n* = 12). E) Demonstration of underwater and dynamic stability of the TX140 hydrogels applied to seal an 8 mm puncture in porcine heart tissue (*n* = 6). The hydrogels remained stable after 24 h soaking in a 37 °C water bath (Movies S1 and S2, Supporting Information). F) live mice subcutaneous injection model that confirms gelation and adhesion of TX140 without any physical containment (*n* = 24).

The main constituent in the formulation of TX140 is PNPHO polymer.^[^ ^39^ ^]^ The method for the synthesis and purification of PNPHO for large scale production has been optimized to acquire high yield, reduce polydispersity, and decrease impurities and residues of organic solvents in the final product. In the current study, PNPHO polymer with 81 mol% NIPPAm, 7 mol% polylactide/‐hydroxy methacrylate, 5 mol% oligo (ethylene glycol) and 7 mol% *N*‐acryloxysuccinimide (Figure S1, Supporting Information) is used based on comprehensive benchtop screening studies to optimize the lower critical solution temperature (LCST) of the polymer and its gelation rate at 37 °C. Importantly, the synthesized PNPHO polymer is purified in water, as opposed to organic solvents, to remove impurities and reduce the polydispersity of the polymer. The chemical structure and molar composition of PNPHO, corresponding ^1^H‐NMR spectra of the polymer and the associated characteristic peaks to all four building blocks are shown in **Figure** **1**A. The thermal characterization of PNPHO showed 1% water content and two degradation processes at 287 and 401 °C (Figure S2, Supporting Information).

Flowability of the polymer solution through a 22G needle was identified as a critical requirement to ensure that the final product is amenable to both superficial‐ and deep‐tissue administration.^[^ ^41^, ^42^ ^]^ To this end, a range of PNPHO solutions in PBS with different polymer concentrations were formed. For quantitative assessments, viscosity‐shear rate characteristics of PNPHO solutions at 37 °C were measured (Figure S3, Supporting Information); the decreasing trend of viscosity with shear rate is indicative of shear‐thinning behavior of PNPHO solution. In addition, in vitro injectability of PNPHO solutions with different polymer concentrations through different gauge needles was assessed blindly with three clinicians (Table S1, Supporting Information). The results showed that 140 mg mL^−1^ of PNPHO is the optimum concentration as the resulting solution conveniently flows through a 22G needle with thumb‐pressure and the corresponding injection force was less than 5N and is therefore within the suitable range for manual injection. After injection and in simulated physiological conditions, the resulting PNPHO hydrogel degrades within 4 days which would deter the intended mechanism of action as a temporary matrix for in vivo tissue repair. To address this shortcoming, *N*‐acryloxysuccinimide functional groups in the chemical composition of PNPHO were used to crosslink this polymer with primary amines of peptide/protein to control the resorption rate of the matrix.

Accordingly, a preliminary screening study was undertaken to identify the ideal peptide/protein component in the formulation of TX140 to assess their ability to bond with succinimide ester groups of PNPHO. The molecular weight of a peptide/protein is known to affect its ability to chemically interact with the functional groups of PNPHO polymers. Small peptides with molecular weight of less than 2 kDa fail to effectively crosslink PNPHO polymer (*M*_w_ of ≈70 kDa) due to the space‐impedance effect of the polymer chain. Conversely, large proteins (greater than 10 kDa), similar to *α*‐elastin in our previous study,^[^ ^39^ ^]^ form nonspecific bonds and thus fail to interact with PNPHO. In addition, the incorporation of biologically active peptides/proteins was intentionally avoided to negate the risks associated with ectopic tissue formation with a view to developing a tissue agnostic injectable hydrogel.^[^ ^7^, ^8^, ^9^ ^]^ The results indicate that Thymosin‐*β*4 is an ideal candidate to crosslink PNPHO as it has a molecular weight of 4.9 kDa and is devoid of any phenotypic biological activity.

To quantify the required amount of Thymosin‐*β*4 to crosslink PNPHO, the chemical interaction between the succinimide ester groups of polymer and amine groups of the peptide was assessed using ^1^H‐NMR method established by Kimhi and Bianco‐Peled.^[^ ^43^ ^]^ The coil to globe transition behavior of the temperature responsive matrices at their LCSTs or higher immobilizes hydrogen ions during ^1^H‐NMR data acquisition and thus diminishes the hydrogen peaks in covalently bonded polymer chains.^[^ ^43^ ^]^ Results in Figure 1B‐i show that peak immobilization is achieved at a noticeably lower temperature when Thymosin‐*β*4 interacts with PNPHO chains. To find the required amount of Thymosin‐*β*4 to crosslink the PNPHO polymer chain, solutions with different Thymosin‐*β*4 concentrations between 10 and 60 mg mL^−1^ were added to 140 mg mL^−1^ PNPHO. Corresponding LCSTs of the resulting PNPHO‐*co‐*Thymosin‐*β*4 solutions were identified by using D_2_O/(m) at different ^1^H‐NMR data acquisition temperatures (Figure 1B‐ii). The intersection between the lines through baseline and the leading edge of the D_2_O/(m) represents the LCST of PNPHO‐*co‐*Thymosin‐*β*4 solutions with different peptide concentrations.

The LCST of the non‐crosslinked PNPHO polymer solution was 24 °C (Figure S4, Supporting Information). However, the results showed that crosslinking of PNPHO polymer with 10 and 20 mg mL^−1^ of Thymosin‐*β*4 decreases the LCST from 24 to 23 °C and 22 °C, respectively. The LCST of PNPHO‐*co*‐Thymosin‐*β*4 solution plateaus with 30 mg mL^−1^ of Thymosin‐*β*4 at 21 °C; further increase of Thymosin‐*β*4 concentration to 60 mg mL^−1^ has no impact on the LCST of the solution (Figure 1B‐iii). Therefore, it was concluded that 30 mg mL^−1^ of Thymosin‐*β*4 is required to crosslink 140 mg mL^−1^ of PNPHO effectively. Any excess amount of Thymosin‐*β*4, e.g., above 30 mg mL^−1^, has no crosslinking role and thus was avoided in the formulation of TX140. An in vitro cell study with Beas‐2B cells showed that free Thymosin‐*β*4 peptide significantly decreased the IL‐6 basal levels, whereas TX140 did not induce any IL‐6 variation on the basal levels (Figure S5, Supporting Information). This result further endorses the physical role of Thymosin‐*β*4 in the formulation of TX140. As expected, the crosslinking effect of Thymosin‐*β*4 increases the structural stability of the matrix in simulated physiological conditions from 4 days to at least 2 weeks, critical to achieving the intended mechanism of action for TX140 as a temporary matrix to support tissue ingrowth and healing.^[^ ^44^ ^]^

### 2.2 Physical Characterization

The gelation kinetics of the hydrogel precursor solution and its adhesivity postadministration are two pivotal physical parameters for the successful application of injectable hydrogels.^[^ ^45^, ^46^, ^47^ ^]^ Accordingly, the gelation and adhesivity of TX140 under three physiological conditions were investigated to assess its potential for a broad range of therapeutic applications. A critical size osteotomy, 8 mm in diameter and 10 mm in depth,^[^ ^48^ ^]^ was used in a live sheep model to examine injectability and adhesion of TX140 in a surgically created bone void. The results in Figure 1C and Movie S1 (Supporting Information) showed the product can be injected into the defect site and it rapidly forms a hydrogel, mixes with blood, stabilizes the clot and fills the cavity. The gelation was achieved despite the presence of active bleeding, which is known to increase the risk of product dislodgment due to dilution of hydrogel constituents. The noticed favorable administration characteristic is attributed to the optimized gelation kinetics of TX140, particularly the role that Thymosin‐*β*4 plays in lowering the LCST of the conjugated system.

The adhesivity of the platform material under dynamic conditions was assessed by injecting TX140 into an ex vivo sheep chondral defect (Table S2, Supporting Information). For this ex vivo model, all limbs were disarticulated from the body at the coxofemoral joint and a standard arthrotomy was made on each limb whilst in flexion to access the medial femoral condyle (MFC) of the femur. A 6 mm diameter and 6 mm deep defect was drilled through the central weight bearing aspect of the MFC and TX140 was injected into the formed defects. As Figure 1D indicates, TX140 remained at the site without dislodgement after the joint was subjected to 100 range of motion. Considering the potential application of the technology for internal wound closure and sealing, the stability and adhesion capability of the TX140 was demonstrated via applying the gels on pig heart tissue maintained at 37 °C in a water bath for 24 h (Figure 1E; Movie S2, Supporting Information). Further, the hydrogel was subjected to water pressure (at ≈37 °C) to assess stability under shear forces. As demonstrated, TX140 resisted the harsh water pressure and showed robust stability in simulated physiological conditions. In addition, an in vivo subcutaneous mouse model was used to investigate the gelation and retention of TX140 hydrogel at a site with no physical containment. In this animal study, 1 mL of TX140 was injected subcutaneously to two sites (2 mL in total per animal). Postadministration, TX140 solution formed an adhesive hydrogel and remained at the injection site without any physical containment (Figure 1E). The 3D structure of the resulting hydrogel was macroscopically detectable for at least 7 days post injection. Gelation and adhesion of TX140 under different conditions are critical attributes of the device thus allowing its application for a wide range of clinical indications and minimizing the risk of device malfunction.

### 2.3 TX140 Quality and Safety Assessments for Medical Applications

A thorough investigation was conducted to assess the safety of TX140 hydrogel. To this end, standard methods were used for determining sterility assurance level (ISO11137), the residues of organic solvents (ICH Q3C (R5)), endotoxin level (BP (XIV C)/Ph Eur (2.6.14), USP/NF 〈85〉), local inflammatory and systemic toxicity (ISO10993‐11, ‐5 and ‐6). In these assessments, a maximum TX140 administration volume of 20 mL per patient was assumed, covering a broad range of potential clinical applications of the product.

In the context of translational medicine, terminal sterilization of implantable medical devices is of great importance for scalability and wide clinical adoption.^[^ ^49^, ^50^ ^]^ Therefore, the compatibility of TX140 with terminal sterilization techniques was investigated. Recognizing that TX140 is a water‐based solution with a glass transition temperature of ≈61 °C (Figure S6, Supporting Information), the product is deemed incompatible with conventional thermal sterilization techniques. Similarly, temperature excursions (above 61 °C) must be avoided during Gamma, X‐ray or E‐beam sterilization. Accordingly, Gamma irradiation on dry ice was explored to investigate the compatibility of TX140 with terminal sterilization using radiation. ^1^H‐NMR and liquid chromatography–mass spectrometry (LC‐MS) assessment of TX140 post treatment on dry ice showed that the gamma‐irradiation (at 40 kGy or less) does not impact the physical and chemical characteristics of the device and its constituents (Table S3, Supporting Information). Accordingly, results showed that TX140 is compatible with gamma irradiation with dry ice to terminally sterilize the matrix with the sterility assurance level of 10^−6^ per ISO11137.

The results of gas chromatography (per USP467) and ^1^H‐NMR analyses showed the residues of organic solvents that are used during the production process (i.e., dimethyl formamide, tetrahydrofuran, ethyl acetate and toluene) of the hydrogel, were 30 to 300‐fold less than the maximum allowable limits (Table S4, Supporting Information). These results confirmed that the purification of PNPHO with water at 30 °C is an effective and safe method for removing organic solvent residues from the product to mitigate the short and long‐term associated safety risks. In addition, endotoxin levels in the final products were measured as it leads to the pyrogenic febrile reaction in patients post administration. Per USP 〈85〉 for medical devices, the endotoxin limit must be equal or less than 20.00 EU per device, except for those medical devices in contact with the cerebrospinal fluid which is beyond the scope of our study. Accordingly, the acceptance criteria for TX140 with the assumed maximum dose of 20 mL per patient is equal to or less than 1.00 EU mL^−1^. The endotoxin levels in TX140 were <0.100 EU mL^−1^,10 fold less than the maximum allowable amount (Table S5, Supporting Information).

**Figure 2:**
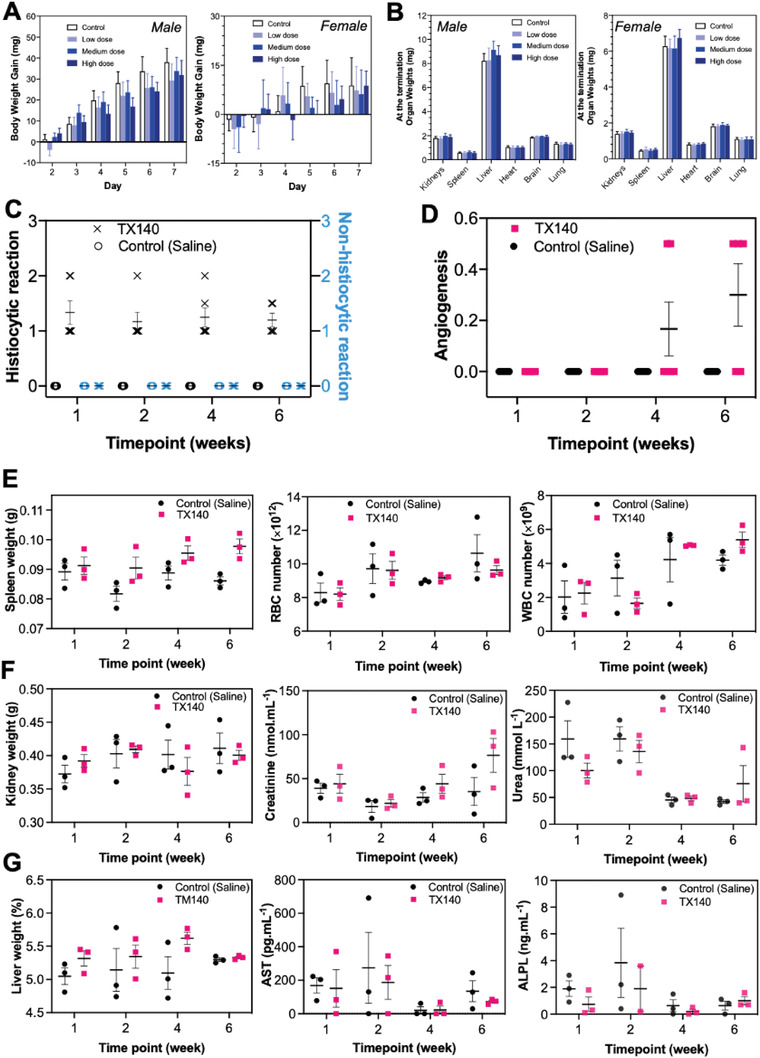
Acute toxicity, local, and systemic biocompatibility assessment of TX140. A) Body weight gain of female and male rats by polar and nonpolar extracts of TX140 during a week for acute toxicity assessment (*n* = 80, 40 female and 40 male animals). B) Organ weights of female and male rats by polar and nonpolar extracts of TX140 at the termination of the acute toxicity assessment (*n* = 80, 40 female and 40 male animals). C) inflammatory response (histiocytic and nonhistiocytic responses) to TX140 and normal saline at different time points. D) local angiogenesis at TX140 and normal saline injection site at different time points. E) spleen weight, red blood cell (RBC) and white blood cell (WBC) counts of TX140 and normal saline injected animals at different time points. F) kidney weight and kidney biochemistry markers (urea and creatinine) of TX140 and normal saline injected animals at different time points. G) liver weight and liver biochemistry markers of TX140 and normal saline injected animals at different time points (aspartate aminotransferase (AST) and alkaline phosphatase (ALP)). *n* = 24 in total, 6 at each time point for measurements reported in (C)–(G).

In accordance with ISO10993‐11, acute systemic toxicity of TX140 was investigated in which 80 mice were administrated via intraperitoneal injection of polar and nonpolar extracts of the product at three dose levels up to 2 mL kg^−1^. Proportionally, this equates to administration of 160 mL of the product per patient which is 8 times higher than the maximum expected clinical dose (20 mL of TX140 per patient). Body weight measurements, hematology, biochemistry, necropsy, and histopathology were performed on animals. The results in **Figure** **2**A showed that there were no significant differences (*p* > 0.05) in body weight gain of animals treated with either +Control (solvents without TX140 extracts) or any of three dose levels of TX140 extracts. The assessment of kidneys, spleen, liver, heart, brain, and lungs of the treated animals also showed that there was no dose dependent impact (*p>*0.05) on the weights of these internal organs (Figure 2B). Comprehensive hematology and biochemistry assessments of all animals treated with three different doses were conducted. Results from hematology and biochemistry parameters of animals injected with polar and nonpolar extracts of TX140 at three dose levels showed that the product did not induce any changes in relation to function of internal organs (Figures S7 and S8, Supporting Information). The histological assessments of internal organs and complete hematology and biochemistry assessments showed no impact on any organs and confirmed the acute systemic biocompatibility of TX140 in accordance with ISO10993‐11.

### 2.4 In Vivo Biocompatibility, Bioresorbability, and Biodegradation of TX140

Long term biocompatibility and bioresorbability of TX140 were assessed to determine TX140 biodegradation pathway by injecting 2 mL of the product (equal to ≈8 wt% of mouse body weight) subcutaneously in a mouse model (male Balb/c, 10 weeks old, *n* = 24). The selected amount represents a significantly greater volume of the product intended to be used in a clinical setting and therefore was selected to exacerbate any potential pathological impacts from the product. General animal health, local and systemic inflammatory response, internal organ function and histology were assessed to determine the biodegradation pathway over 6 weeks. The outcomes were compared with a 2 mL normal saline injection (+Control). The observed trend in animal weight gain treated with TX140 was comparable to the +Control group (normal saline injection) at different time points (Figure S9, Supporting Information). Macroscopic assessment of the administration sites demonstrated that the product was fully resorbed within 2–4 weeks post administration. This finding was further endorsed by results from the histological evaluations of the injection site at two and four weeks post administration of TX140. H&E assessments of the sites showed the presence of small pools of hydrogel network in the range of ≈10–15 µm in diameter at areas closer to epidermis at 2 weeks, while no TX140 hydrogel structure was visible after 4 weeks. The results from macroscopic assessments and histological evaluations confirmed that TX140 was bioresorbable within four weeks post administration.

Concerning local inflammatory response to TX140 hydrogels, Hematoxylin and Eosin (H&E) assessment of dermal injection sites displayed no sign of inflammatory neutrophils, eosinophils, and lymphocytes (Figure 2C). This result confirmed that TX140 does not induce any nonhistiocytic inflammation. As expected, histiocytes were present in the vicinity of the injection site, which could either be due to oedema generated by the hydrophilic nature of the gel and/or the gel product itself being phagocytosed as part of the wound healing response to initiate tissue remodeling. In relation to neo‐blood vessel formation, angiogenesis was observed in animals injected with TX140 (Figure 2D) at week 4 and week 6 postinjection. Conversely, no capillary structures were observed in the animals injected with normal saline. Combined angiogenesis and histiocytic presence at the local injection site of TX140 is suggestive of neo‐tissue formation and remodeling, particularly in weeks 4 and 6 when angiogenesis was more frequently observed.

In vitro studies showed that the cleavage of PLA from the PNPHO network leads to increase of LCST of TX140 and subsequently the gradual liquification of the hydrogel at physiological conditions.^[^ ^39^ ^]^ It is hypothesized that this effect ultimately leads to TX140 excretion and thus the impact of the product resorption was assessed systemically to confirm the safety of the breakdown components and the associated degradation pathway(s) of the product. The systemic inflammatory response showed no significant difference between the white blood cell (WBC) and red blood cell (RBC) count in animals injected with TX140 and normal saline at different time points (Figure 2E). Spleen weight was not significantly different and histological analysis of spleen tissue of animals injected with TX140 and normal saline showed no splenic pathology (Table S6, Supporting Information). The combined results in Figure 2E from WBC/RBC, spleen weight and histopathology assessment of animals treated with TX140 do not suggest any splenic pathology up to 6 weeks post administration and demonstrate full resorption of the product. Similarly, the combined results in Figure 2F of kidney weight, creatinine and urea levels and kidney histological analysis showed that the bioresorption of TX140 and its breakdown products have no pathological impact on renal systems. Similarly, the combined results in Figure 2G confirmed no hepatic impact from the bioresorption and biodegradation; this was concluded based on no significant changes in percentage liver weight to body weight, liver function enzymes (Aspartate transaminase, AST and Alkaline Phosphatase, ALPL) and liver histology assessments. Mild to moderate granulomatous inflammation, defined by histiocytic presence was detected in mouse lungs at 4 weeks and 6 weeks post TX140 injection, suggestive of a biodegradation pathway that is commonly observed in subcutaneously implanted foreign material. The mild to moderate degree of granulomatous formation is attributed to excessive dosage of TX140 used in the study (8 wt% of body weight which is significantly higher than the intended 20 mL per patient). No material remnants were found in lung histopathology and general mouse health was not affected by the mild to moderate granuloma presence.

### 2.5 Soft‐Tissue Repair and Angioconductive Properties of TX140

**Figure 3:**
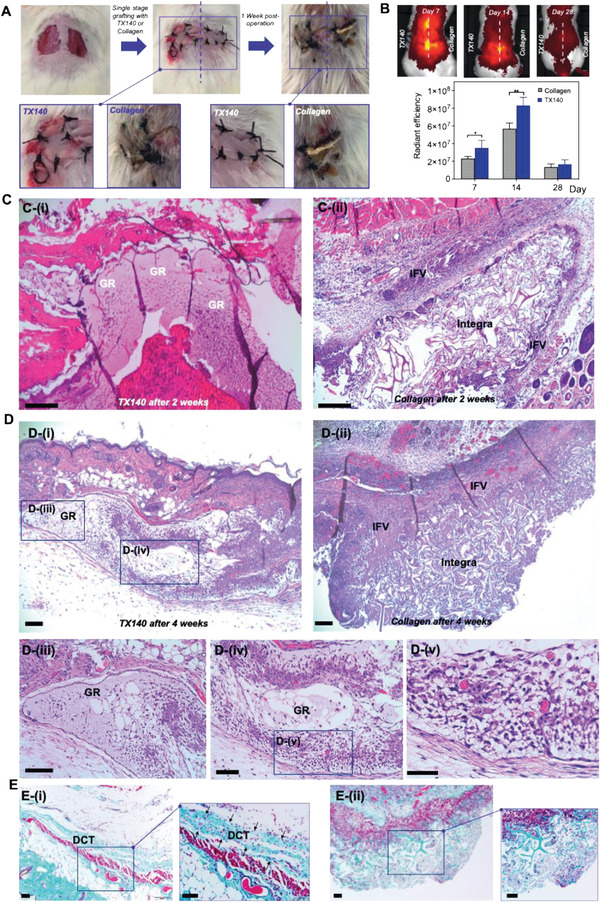
Soft‐tissue healing and angioconductive properties of TX140. A) Intraoperative wounds after harvesting full‐thickness skin grafts and grafting, treated with TX140 (left) and commercially available collagen scaffold as the gold standard (right) at day 0 and day 7 post skin grafting (*n* = 27). B) Radiant efficiency of TX140 and collagen scaffold in a murine model after 7, 14, and 28 days (*n* = 6, statistical significance * for *p* < 0.05 and ** for *p* < 0.01)). C) H&E stained cross‐sections of TX140 i) and collagen scaffold ii) after 14 days. D) H&E stained cross‐sections of TX140 i) and the collagen scaffold ii) after 28 days. Cross sections of TX140 iii–v). E) MT stained cross‐sections of Integra and TX140 i) and collagen scaffold ii) biopsies 28 days postskin grafting. GR: TX140 hydrogel residues and IFV: inflammatory fibrosis. DCT: dermal connective tissue. The scale bar in all panel is 100 µm. For (C)–(E) *n* = 27 in total and 12 at each time point.

Cell infiltration and vascular ingrowth are two important parameters for the selection of a biomaterial for tissue regeneration. To this end, neovascularization and host cell infiltration within TX140 hydrogel were compared with a commercially available collagen scaffold in a full thickness dermal mouse model (Table S7, Supporting Information). The selected study provides a model to investigate the capability of TX140 to integrate with host tissues and compare the outcome with a well‐understood and naturally derived dermal matrix as a positive control.^[^ ^51^ ^]^ The selected collagen scaffold in this study was glutaraldehyde cross‐linked dermal template that is commonly used in dermal reconstructive surgeries. As shown in **Figure** **3**A, two identical full‐thickness dermal defects were surgically created in each animal and the overlying skin from the defects used as autologous skin grafts. The defects were treated with either TX140 or the collagen scaffold and covered with the harvested skin graft from the opposite site (single stage, full‐thickness defatted skin graft). The collagen scaffold was required to be cut‐to‐size to be able to cover the wound bed, whereas TX140 was directly applied to the site and formed an adhesive hydrogel to fully covered the full site.

No partial or full graft failure was noted in TX140 treated site. Conversely, in the collagen scaffold treated group, the graft take rate was ≈80% with partial or full skin graft failures observed in some animals by the detachment of the grafted tissue from the surgery site (Figure 3A). Neovascularization at the treatment sites was quantified by in vivo fluorescent radiant efficiency of the animals. As depicted in Figure 3B, the fluorescent radiant efficiency of TX140 treated sites after 7 days (*p* < 0.05) and 14 days (*p* < 0.01) postoperation was significantly higher than that of sites treated with collagen scaffolds. Therefore, based on the results in Figure 3B, it was concluded that TX140 expedites neovascularization at an early stage and thus provides the grafted site with hematological supply to integrate with the host tissue. On day 28, fluorescent radiant efficiency showed that neovascularization at the site was stabilized to allow tissue maturation and subsequent progression of wound healing.

The underlying mechanism of action and cellular interaction with TX140 was further examined using histological analysis. H&E staining showed that, as expected, the minimal inflammatory response was observed around TX140 after 2 weeks postoperation. A thin layer of fibrotic tissue surrounded TX140 residues (GR in Figure 3C‐i). Conversely, sites treated with the collagen scaffold showed multilayered inflammatory fibrous tissue (IFV in Figure 3C–ii) at 2 weeks postoperation, suggesting an inflammatory driven encapsulation of the construct from the host tissue. Therefore, the ability of the host progenitor cells to permeate within the collagen scaffold was limited by the presence of fibrotic tissue and hence the noted full/partial graft failure in animals treated with the collagen membrane. Similarly, after 4 weeks, the inflammatory response to collagen scaffolds was significantly higher than to TX140 (Figure 3D–i,ii). As expected, in TX140 treated groups, the H&E stained cross‐sections showed abundant infiltration of host cells to progress skin remodeling (Figure 3D–iii,iv)) and blood vessel ingrowth (Figure 3D‐v). In addition, results from Masson's Trichrome (MT) staining confirmed that the formation of native collagen fibers (dermal connective tissue, DCT in Figure 3E‐i) within the structure of TX140 was significantly higher than that detected within the structure of the control group (Figure 3E–ii). This result further reaffirms the ability of TX140 to support host cell infiltration.

### 2.6 Hard‐Tissue Repair with TX140

This part of the study aimed to investigate the potential of TX140 to support hard tissue repair by providing a cell permeable matrix. Critical size femoral and humorous osteotomies (8 mm diameter × 10 mm depth) were formed in a sheep model.^[^ ^48^ ^]^ Three treatment groups were investigated, including no treatment (empty defect −Control), TX140 and iliac crest autologous bone graft (+Control). Bone healing at the site was histologically assessed 6 weeks and 12 weeks post operation. Product usability in the osteotomy model and its efficacy to integrate with bony tissue were investigated based on predefined acceptance criteria (Table S8, Supporting Information).

**Figure 4:**
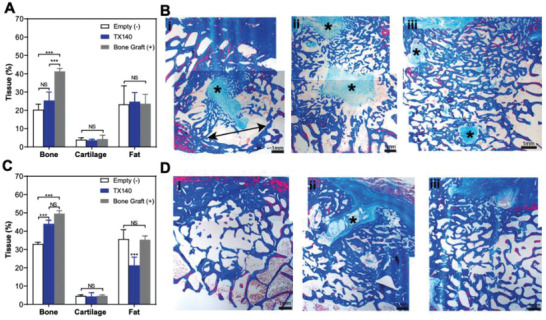
Hard‐tissue healing in sheep osteotomy model. A) Bone, cartilage, and fat formation in osteotomy sheep model after 6 weeks. B) Masson's Trichrome (MT)‐stained decalcified sections of lesion explants from i) Empty, ii) TX140, and iii) bone graft groups after 6‐weeks postsurgery. C) Bone, cartilage, and fat formation in an osteotomy sheep model after 12 weeks postsurgery D) MT‐stained decalcified sections of lesion explants from i) Empty, ii) TX140, and iii) bone graft groups after 12‐weeks postsurgery. The scale bar in all panels is 1 mm. Asterisks highlight the new bone formation foci of fibrous connective tissue (*n* = 6).

H&E staining of the treatment sites showed that the inflammatory responses to TX140 were relatively lower than the sites treated with autologous bone graft at 6 weeks (Figure S10, Supporting Information) and 12 weeks postoperation ((Figure S11, Supporting Information). TX140 and +Control sites contained residual inflammatory foci mostly comprising foamy macrophages with lymphocytes associated with small foci of partially hydrolyzed TX140 and autologous bone graft respectively. The results in **Figure** **4**A display the extent of bone, cartilage, and fat tissue content after 6 weeks post operation in defects treated with different groups. Masson's Trichrome (MT) stained, decalcified sections of the lesion after 6‐weeks postsurgery demonstrated that animals with empty defects (−Control) showed large residual spaces, devoid of mesenchymal tissue (double‐headed arrow in Figure 4B‐i). As expected, TX140 treated sites (Figure 4B‐ii) showed similar, but slightly more new bone formation within the treatment sites than −Control at the 6‐week time point. Sites treated with autologous bone graft showed significantly higher new bone formation within the defect (Figure 4B‐iii) than both groups at 6 weeks postoperation. This is characterized by anatomizing bone trabeculae interspersed via medullary adipose tissue and a few small foci of fibrous connective tissue (asterisks in Figure 4B‐iii). After 12 weeks, the results in Figure 4C confirmed that TX140 and autologous bone graft treated groups showed similar (*p* > 0.05) healing. The extent of healing in TX140 compared to −Control was significantly more pronounced (*p* < 0.001). The latter finding is further endorsed by results in Figure 4D‐i, as the −Control sites were predominantly comprised of thin anatomizing bone trabeculae with well‐differentiated pluricellular medullary adipose tissue. Notably, the sites treated with TX140 and autologous grafts showed the presence of compact and trabecular woven bone with a few residual islands of fibrovascular connective tissue (asterisk in Figure 4D‐ii,iii). Therefore, the extent of bone regeneration in TX140 and autologous graft treated sites were comparable after 12 weeks.

### 2.7 Clinical Application of TX140 for Soft and Hard Tissue Repair

**Figure 5:**
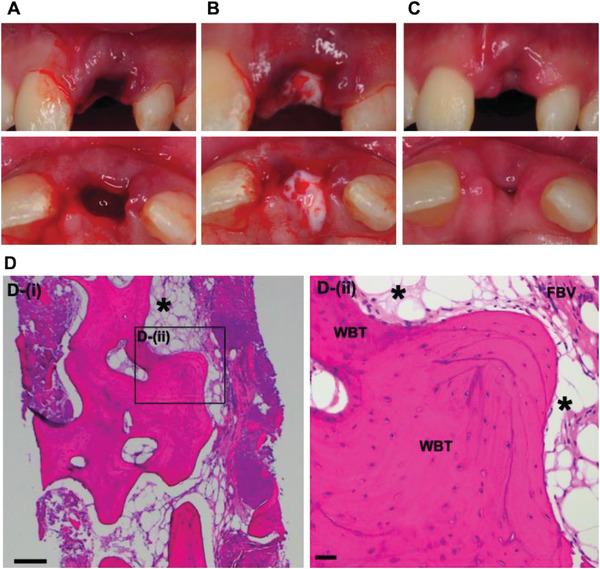
Clinical study of TX140 post tooth extraction. A) Extraction site with active bleeding, B) in situ gel formation of TX140 in extraction site, adhesion and mixing with blood at the injection site without the need for primary closure C) soft tissue healing and wound closure at TX140 treated site 7 days post operation (*n* = 10). D) MT stained TX140 treated site 3 months postoperation, product residues (asterisk), woven bone trabecular (WBT) and fibrovascular tissue (FBV). The scale bar in panel (D) is 200 µm (*n* = 9).

The results from in vivo studies in small and large animal models confirmed the safety and effectiveness of TX140 for soft and hard tissue regeneration. Subsequently, a ready‐to‐use configuration of TX140 was clinically tested in a single‐armed, pilot investigation on 10 healthy participants in which the product was injected to the base of a maxillary mandibular socket after tooth extraction (**Figure** **5**A). Participants’ demographic information is summarized in Table S9 (Supporting Information) and the trial was independently monitored in compliance with ISO 14155. All participants had planned implant placement allowing biopsy collection and histological evaluations of the sites at 3 months post extraction to assess TX140 interactions with soft and hard tissue.

The principal investigator could readily inject TX140 into the base of the socket through a 22G needle. The product was administered to all 10 participants and formed a hydrogel post administration (Figure S12, Supporting Information). Subsequently, due to the hydrophilic nature of the product, TX140 hydrogel absorbed blood at the site and stabilized the clot. This clinical study also demonstrated that TX140 adhered to surrounding tissue and it was not necessary to use membranes and microsuturing to stabilize the site post product administration (Figure 5B). Hence, the process was more convenient and less time consuming than the current common practice that involves physical containment (suture), primary closure and the use of membranes at the socket site. No adverse effects (e.g., infection, inflammation, pain or discomfort) were reported during the follow up visit of any participant (Table S10, Supporting Information). These results endorsed the safety of TX140 hydrogel as an implantable device.

The treatment site with TX140 involved both soft and hard tissue and therefore provided a unique opportunity to assess the tissue agnostic nature of the product to support healing. The results in Figure 5C showed the extent of soft tissue healing and subsequent wound closure after one‐week post treatment with TX140. Participants underwent a planned implant placement procedure three months after tooth extraction and treatment with TX140. At this point, biopsies were collected from the injection site; H&E and MT stains were used to analyze the behavior of TX140. There was no evidence of necrosis, foreign body type giant cell or foreign body reactions to TX140. These findings confirmed that TX140 is well‐tolerated and the product was predominantly resorbed as only small pools of the hydrogel were detected (Figure 5D, asterisk). A comprehensive summary of findings from the histological evaluations of the sites showed active bone remodeling and viable trabecular bone formation and the presence of osteoblasts and osteoclasts (Table S11, Supporting Information). MT section of the treatment site with TX140 showed the formation of interconnected viable bony trabeculae, a mixture of woven, lamellar bone and active osteoblasts and osteoclasts (Figure 5D).

## 3 Discussion

Recent advancements in minimally invasive surgeries provide a unique opportunity for clinical adoption of tissue agnostic injectable hydrogels as temporary cell permeable matrices to expedite healing and ultimately improve patients’ health outcomes.^[^ ^52^, ^53^ ^]^ Despite the plethora of injectable systems that are commercially available and in development, widespread clinical adoption of injectable hydrogels remains low; device malfunction due to cumbersome preparation steps, low adhesion, and severe inflammatory responses are the main limiting factors.^[^ ^2^, ^54^, ^55^, ^56^ ^]^ TX140 injectable hydrogel has shown to address many of these unmet clinical needs. The product comprises a synthetic polymer (PNPHO) and a synthetic peptide (Thymosin‐*β*4) that work synergistically to achieve the intended mechanism of action as a flowable scaffold to support tissue agnostic regeneration.

The results from the injectability and adhesivity of TX140 into different anatomic sites showed that the optimized gelation kinetics of TX140 facilitate its administration into deep tissues without clogging the needle or dislodgment of the hydrogel at the site. This specific property is of great importance compared with previously developed hydrogels that have unfavorable gelation kinetics and consequently poor structural stability and retention.^[^ ^1^, ^25^, ^30^, ^57^, ^58^, ^59^ ^]^ The favorable gelation kinetics of TX140 is attributed to the optimized PNPHO chemical composition and the crosslinking effect of Thymosin‐*β*4, which decreases the LCST of the hydrogel from 24 to 21 °C. The chemical interaction between PNPHO and Thymosin‐*β*4 prolongs the bioresorption time of TX140 to 2–12 weeks (depending on the anatomical site of administration), which is sufficient to support the healing cascade for a wide range of indications and thus further substantiates the physical role of Thymosin‐*β*4 in the formulation of TX140.

It was hypothesized that TX140 can be used as a tissue agnostic injectable system for soft and hard tissue regeneration by providing a cell permeable adhesive hydrogel at the site to allow ingrowth of host progenitors cells. Unlike previously developed tissue specific polymer‐peptide hydrogels,^[^ ^60^ ^]^ TX140 does not have any phenotypic properties, a pivotal requirement for a tissue agnostic biomaterial. This strategy was implemented to expedite future clinical adoption of TX140 by avoiding device complications associated with ectopic tissue formation^[^ ^7^, ^8^, ^9^ ^]^ thereby streamlining the device's regulatory approval process with a single chemical composition.^[^ ^61^ ^]^ The specific chemistry and composition of TX140 mitigate the limitations regarding local inflammation and systemic toxicity which are widely reported in the use of current injectable and synthetic polymer‐peptide systems.^[^ ^62^, ^63^ ^]^ The residues of organic solvents in TX140 are negligible and well below the limits specified by international standards, this is a key requirement to preserve the viability of cells and the natural regenerative capability of host tissues.^[^ ^64^ ^]^ In addition, the level of endotoxins in TX140 was minimized as they are known to directly contribute to the inflammatory process and upregulate inflammatory mediators, including TNF‐*α*, IL‐1, and IL‐6.^[^ ^65^ ^]^

Biocompatibility and biosorption of TX140 were investigated by full hematological and histopathological assessments of animals treated with an ultrahigh dose of TX140. In the histopathological assessment of all internal organs and the lungs displayed mild to moderate granulomatous inflammation in animals at 4 weeks and 6 weeks post TX140 injection. The noticed moderate inflammatory response in lungs was expected due to the ultra‐high volume of the product used and its chemical composition. Specifically, PNPHO is partially based on polylactide (7–8 mol%) which breaks down into lactic acid which oxidizes in tissues to carbonic acid and is excreted via the lungs.^[^ ^66^ ^]^ In a previous study, polyurethane implantable scaffolds demonstrated lung redness and granulomatous inflammation while using a significantly reduced amount of implanted product^[^ ^67^ ^]^ compared to the volume of TX140 in this study. Recognizing that TX140 was fully resorbed within 2–4 weeks post subcutaneous injection, even at ultrahigh doses, histopathological assessments of liver, kidney and spleens, their weights and relevant blood markers (AST, ALPL, creatinine, urea, WBC, and RBC count) confirmed that full biosorption of TX140 does not impact any other internal organs involved in excretory pathways.

In a full thickness dermal defect animal study, TX140 provided a cell permeable matrix which was not impeded by fibrosis around the structure; the thickness of fibrotic tissue was less than 2–3 cell layers on average, which was significantly less than that found around a collagen based dermal matrix (Integra after removing the silicon barrier for this single step skin grafting study). Despite the synthetic nature of TX140, the results from the dermal defect mode were contrary to previous studies in which the inflammatory response to synthetic scaffolds was reported to be more pronounced compared to collagen scaffolds.^[^ ^68^, ^69^ ^]^ TX140 allowed cell ingrowth and neovascularization at the defect site which provided the grafted site with hematological supply for complete tissue integration and graft stabilization. The results therefore confirmed that TX140 acts as a physical scaffold to support the regeneration of dermal connective tissue as intended.

The scaffolding effect of TX140 was also evident when the product was applied into a critical size bone defect. As expected, due to the lack of osteoinductive or phenotypic properties of TX140, the healing was less evident in the early stages post trauma than in the sites treated with osteoinductive autologous grafts. However, 12 weeks postoperation, the healing at TX140 treated sites was superior (*p <* 0.05) to sites with no treatment and similar (*p* > 0.05) to osteoinductive iliac crest grafted sites. These results suggest that in the early stage, the infiltrated progenitor cells within TX140 display limited osteogenic activity but over time, the scaffolding properties of TX140 provides an optimum environment for cell proliferation, maturation, and subsequent calcification. In contrast, in the absence of osteogenic compounds or osteoconductive ceramics, previous studies using synthetic and thermoresponsive hydrogels showed negligible bone regeneration with the use of poly(glycolic acid and poly (*α*‐hydroxy acid) based products or woven surgical meshes.^[^ ^61^ ^]^ The lack of bone integration with these synthetic scaffolds was attributed to mild inflammatory responses at the sites and the lack of cell adhesion, both of which impede the intended mechanism of action. The preclinical use of TX140 in dermal and critical size bone defects confirmed that the physical scaffolding nature of the hydrogel provides a temporary matrix to support both soft and hard tissue regeneration.

The clinical usability and efficacy of TX140 for soft and hard tissue regeneration were investigated with the administration of the product to the base of a socket post tooth extraction. The results showed that TX140 is adhesive, able to fill the defect entirely, mixes with blood and remains at the site despite active bleeding. These outcomes confirmed TX140 overcomes the main limitations of previously developed systems which are unable to adhere/retain in hydrated environments, particularly in dental, oral and periodontal surgeries.^[^ ^70^, ^71^ ^]^ The innate adhesive nature of TX140 and its gelation at the base of the socket negates the need for primary closure, which further underpins the advantage of TX140 for clinical applications. TX140 promote soft tissue formation as wound closure was achieved after one week with no reports of pain/discomfort in all treated clinical participants. Conversely, in a pilot clinical trial using a commercially available collagen scaffold, complete wound closure and re‐epithelization were reported between 15 and 30 days postsurgery,^[^ ^72^ ^]^ which is notably longer than that achieved using TX140. In addition, the histochemical analyses of the local sites after three months of treatment with TX140 confirmed that the scaffolding nature of the product supports infiltration of local osteoblasts and osteoclasts and thus allows the progression of the healing cascade toward bone remodeling. The results from the pilot clinical study endorse the superior properties of TX140 as an injectable system for tissue agnostic regeneration to address multiple unmet clinical needs.

## 4 Conclusion

The clinical potentials of TX140 as a tissue agnostic injectable hydrogel for soft and hard tissue regeneration were verified in multiple preclinical investigations and a clinical pilot study. The overarching mechanism of action of TX140 is based on the product's unique combination of physical and biological characteristics. The injectable and adhesive nature of the TX140 allow its administration at an injury site without detrimentally impacting the natural regenerative capability of the host tissue. The biocompatibility of the product leads to formation of a very thin layer of fibrotic tissue around the hydrogel and therefore allows infiltration of the host progenitor cells. The gradual and safe bioresorption of the product allows tissue regeneration and neovascularization to support body's innate healing capability to support body's innate healing capability. These attributes are translational to ultimately address multiple unmet needs in different clinical settings.

## 5 Experimental Section

### Synthesis of PNPHO Polymer and Characterization

The complete list of chemical and reagents used in the synthesis of PNPHO and formulation of TX140 are provided in Supporting Information. The method for synthesis of PNPHO was outlined by Fathi et al.^[^ ^39^ ^]^ However, the process was optimized to achieve stoichiometric polymerization, reduce polydispersity, and decrease residues of organic solvents in the final product, all of which are essential for translatability of the final product. Briefly, a solution containing *N*‐isopropylacrylamide (NIPAAm, 81 mol%), *N*‐acryloxysuccinimide (NAS, 7 mol%), polylactide/‐hydroxy methacrylate (PLA/HEMA, 7 mol% with lactate number of 5), and oligo (ethylene glycol (OEGMA, 5 mol%) were fully dissolved in dimethyl formamide (DMF) with a solid content of 16 w/v%. The resulting solution was purged with nitrogen for 20 min at ambient temperature to deoxygenate the reactant solution. The reaction temperature was then increased to 70 °C, at which 0.5 mol% of 4,4′‐Azobis (4‐cyanopentanoic acid) was added to initiate the reaction, under a nitrogen blanket to prevent side chain reactions and oxidation of reactants. The reaction was continued for ≈18 h at 70 °C at 300 rpm. The resulting polymer solution in DMF was purified in sterile water for injection at 30 °C as an antisolvent, followed by lyophilization. The resulting white powder/foam is PNPHO polymer. The polymer is chemically and physically characterized with multiple standard methods (Supporting Information).

### Formulation of TX140 and Characterization

Initially, 30 mg mL^−1^ Thymosin *β*4 solution in PBS was formed. The resulting peptide solution was added to PNPHO to achieve the final polymer concentration of 140 mg mL^−1^. The solution was placed in 2–8 °C under static condition for 24 h to dissolve and conjugate PNPHO and Thymosin *β*4. The resulting single‐phase solution was TX140 and was stored refrigerated for further use. TX140 was assessed based on multiple standard techniques for in vitro, in vivo, and clinical evaluations (Supporting Information). TX140 was terminally sterilizable via Gamma irradiation in dry ice at 25–40 kGy.

### Experimental Design

The current study involved multiple original investigations, all of which were completed based on one‐factorial design with predefined objectives, inclusion/exclusion criteria, sample size, primary/secondary endpoint, and acceptance criteria in accordance with ISO13485 requirements for design and development of medical devices. The associated quality management system was certified and audited by British Standard Institute (certification number MD659261). In relation to physicochemical characterization studies, the production process and findings were validated in accordance with ISO13485, using 10 samples (or greater) from three replicated batches (separate manufacturing, minimum *n* = 30 in total) to achieve 99% probability of conformance and confidence level of 99%. Endotoxin level was completed per FDA guidance (USP 〈85〉) and British Pharmacopeia, Appendix XIV C to define the number of samples, acceptance criteria and methodology. Preclinical in vitro and in vivo biological evaluations were completed based on predefined protocols, samples size (*n* = 6 or greater from 3 replicates, batches) and acceptance criteria by using methods in accordance with ISO10993 or previously published studies. All animal studies were completed after attaining ethic approvals from relevant authorities (University of Queensland, Animal Ethics Committee for acute toxicity study, TETRAQ/078/18/M/427/17 and sheep femoral defect, AE42961; NSW‐Health District, Royal Prince Alfred Hospital Ethics for subcutaneous injection and local inflammatory studies in mice, 2017/026C and 2021/021/3.7). The clinical investigation was a single‐armed (not controlled, not powered and not randomized) pilot clinical trial which was completed based on a predefined clinical investigation protocol (CIP) and with 100% data point monitoring via an independent clinical research organization based on ISO 14155. The clinical study was completed after acquiring human ethics approval from St. Vincent Hospital, Melbourne Australia, HREC/16/SVHM/258. All participants in the clinical investigation were treated after providing informed consent.

### Statistical Analysis

Statistical analysis was conducted using one‐ or two‐way ANOVA, followed by Tukey's multiple comparisons using GraphPad Prism version 9. Quantitative data were expressed as means ± standard deviations and *P* < 0.05 was defined as the significant level for all statistical analyses. The quantified chemical and physical characterizations are based on 99% probability of conformance and confidence level of 99%.

## Conflict of Interest

A.F., T.A., S.M., and J.M. are employed by Tetratherix. A.F. and F.D. are co‐inventors in AU2016301103; EP16829490.8; JP2018‐503480; US15/746810; U2016314146; EP344304; JP2018‐529692; US 17/090078; EP 2794701; US9546235. A.F., D.C., and T.A. are co‐inventors in PCT/AU2020/051332 and Chinese national phase patent application (2020800006185.0). D.C. is a clinical advisor to Tetratherix. While the material in the current study has not been published, the main authors have previously invented and disclosed information pertaining to a family of polymers as outlined in granted patents WO 2013/091001; WO 2017/035587; WO 2017/015703; WO 2021/119727. All other authors declare no competing interest.

## Author Contributions

D.C. and A.F. contributed equally to this work. D.C. performed co‐conceptualization (with A.F.), methodology (clinical trial), validation (clinical trial), writing – original draft. A.F. performed co‐conceptualization, methodology (benchtop testing, preclinical studies, and synthesis/product formulation), writing – original draft, investigation (benchtop testing and synthesis), formal analysis, funding acquisition and supervision. F.O. performed writing – review and editing and visualization. S.M. designed, developed, performed methodology (analytical techniques), investigation (benchtop testing and analytical assessments), review and editing. T.A. performed writing‐original draft and project administration. Y.W. performed methodology and investigation (soft‐tissue repair and angio‐conductive properties). J.M. performed writing – review and editing, methodology and investigation (in vivo biocompatibility and bioresorbability assessment). I.C.C. and V.A.M. performed review, editing and assessment of histopathology (in vivo biocompatibility and bioresorbability assessment), K.L. performed supervision histopathology (in vivo biocompatibility and bioresorbability assessment). P.M. performed supervision (in vivo biocompatibility and bioresorbability assessment and soft‐tissue repair and angio‐conductive properties). W.C. performed supervision and writing – review and editing. B.J.A. performed methodology, review, editing and assessment (in vivo hard tissue preclinical studies). N.L.D. performed investigation (in vivo hard tissue preclinical studies). M.S. performed investigation (analytical assessments and in vitro cell study). P.M.Y. performed supervision (analytical assessments and in vitro cell study). D.T. performed writing – review and editing. H.X.O. performed formal analyses (analytical and biocompatibility investigation) and writing – review and editing. R.S.M. performed investigation (rheology, mechanical testing and ex vivo adhesion study). A.K. and F.D. and performed supervision and writing – review and editing.

## Supporting information

Supporting Information

Supplemental Movie 1

Supplemental Movie 2

## Data Availability

The data that support the findings of this study are available on request from the corresponding author. The data are not publicly available due to privacy or ethical restrictions.

## References

[1] Y. Sun , D. Nan , H. Jin , X. Qu , Polym. Test. 2020, 81, 106283.

[2] F. Rizzo , N. S. Kehr , Adv. Healthcare Mater. 2021, 10, 2001341. 10.1002/adhm.202001341 33073515

[3] H. J. Busscher , H. C. van der Mei , G. Subbiahdoss , P. C. Jutte , J. J. A. M. van den Dungen , S. A. J. Zaat , M. J. Schultz , D. W. Grainger , Sci. Transl. Med. 2012, 4. 10.1126/scitranslmed.3004528 23019658

[4] X. Ren , M. Zhao , B. Lash , M. M. Martino , Z. Julier , Front. Bioeng. Biotechnol. 2019, 7, 469. 32039177 10.3389/fbioe.2019.00469 PMC6985039

[5] E. J. Carragee , E. L. Hurwitz , B. K. Weiner , Spine J. 2011, 11, 471. 21729796 10.1016/j.spinee.2011.04.023

[6] J. Shen , A. W. James , J. N. Zara , G. Asatrian , K. Khadarian , J. B. Zhang , S. Ho , H. J. Kim , 2013, 19, 2390. 10.1089/ten.tea.2012.0519 PMC3807546 23758588

[7] D. Robla Costales , L. Junquera , E. García Pérez , S. Gómez Llames , M. Álvarez‐Viejo , Á. Meana‐Infiesta , J. Cranio‐Maxillofacial Surg. 2016, 44, 1743. 10.1016/j.jcms.2016.08.005 27618716

[8] D. A. Wong , A. Kumar , S. Jatana , G. Ghiselli , K. Wong , Spine J. 2008, 8, 1011. 18037352 10.1016/j.spinee.2007.06.014

[9] P. S. Brannan , R. G. Gaston , B. J. Loeffler , D. R. Lewis , J. Hand Surg. Am. 2016, 41, 602. 27013317 10.1016/j.jhsa.2016.01.013

[10] N. S. Kehr , E. A. Prasetyanto , K. Benson , B. Ergün , A. Galstyan , H.‐J. Galla , Angew. Chem., Int. Ed. 2013, 52, 1156. 10.1002/anie.201206951 23203726

[11] O. Chaudhuri , L. Gu , D. Klumpers , M. Darnell , S. A. Bencherif , J. C. Weaver , N. Huebsch , H. Lee , E. Lippens , G. N. Duda , D. J. Mooney , Nat. Mater. 2016, 15, 326. 26618884 10.1038/nmat4489 PMC4767627

[12] M. G. Kim , T. W. Kang , J. Y. Park , S. H. Park , Y. B. Ji , H. J. Ju , D. Y. Kwon , Y. S. Kim , S. W. Kim , B. Lee , H. S. Choi , H. B. Lee , J. H. Kim , B. Y. Lee , B. H. Min , M. S. Kim , Mater. Sci. Eng. C 2019, 103, 109853. 10.1016/j.msec.2019.109853 31349513

[13] D. W. Lim , D. L. Nettles , L. a Setton , A. Chilkoti , Biomacromolecules 2007, 8, 1463. 17411091 10.1021/bm061059m PMC2562452

[14] J. Wu , Z.‐G. Su , G.‐H. Ma , Int. J. Pharm. 2006, 315, 10.1016/j.ijpharm.2006.01.045. 16616819

[15] H.‐D. Wu , J.‐C. Yang , T. Tsai , D.‐Y. Ji , W.‐J. Chang , C.‐C. Chen , S.‐Y. Lee , Carbohydr. Polym. 2011, 85, 318.

[16] S. Jiang , S. Liu , W. Feng , J. Mech. Behav. Biomed. Mater. 2011, 4, 1228. 21783131 10.1016/j.jmbbm.2011.04.005

[17] D. Gupta , C. H. Tator , M. S. Shoichet , Biomaterials 2006, 27, 2370. 16325904 10.1016/j.biomaterials.2005.11.015

[18] C. Tonda‐Turo , S. Gnavi , F. Ruini , G. Gambarotta , E. Gioffredi , V. Chiono , I. Perroteau , G. Ciardelli , J. Tissue Eng. Regener. Med. 2017, 11, 197. 10.1002/term.1902 24737714

[19] T. M. D. Le , H. T. T. Duong , T. Thambi , V. H. Giang Phan , J. H. Jeong , D. S. Lee , Biomacromolecules 2018, 19, 3536. 30005160 10.1021/acs.biomac.8b00819

[20] E. Ruel‐Gariépy , J.‐C. Leroux , Eur. J. Pharm. Biopharm. 2004, 58, 409. 15296964 10.1016/j.ejpb.2004.03.019

[21] Y. Piao , H. You , T. Xu , H.‐P. Bei , I. Z. Piwko , Y. Y. Kwan , X. Zhao , Eng. Regener. 2021, 2, 47.

[22] H. Samadian , H. Maleki , Z. Allahyari , M. Jaymand , Coord. Chem. Rev. 2020, 420, 213432.

[23] B. Sharma , S. Fermanian , M. Gibson , S. Unterman , D. A. Herzka , B. Cascio , J. Coburn , A. Y. Hui , N. Marcus , G. E. Gold , J. H. Elisseeff , Sci. Transl. Med. 2013, 5, 167ra6. 10.1126/scitranslmed.3004838 PMC3972413 23303605

[24] A. M. Kloxin , A. M. Kasko , C. N. Salinas , K. S. Anseth , Science 2009, 324, 59. 19342581 10.1126/science.1169494 PMC2756032

[25] A. T. Hillel , S. Unterman , Z. Nahas , B. Reid , J. M. Coburn , J. Axelman , J. J. Chae , Q. Guo , R. Trow , A. Thomas , Z. Hou , S. Lichtsteiner , D. Sutton , C. Matheson , P. Walker , N. David , S. Mori , J. M. Taube , J. H. Elisseeff , Sci. Transl. Med. 2011, 3, 93ra67. 10.1126/scitranslmed.3002331 PMC4652657 21795587

[26] H. Lee , S. Chung , M.‐G. Kim , L. P. Lee , J. Y. Lee , Adv. Healthcare Mater. 2016, 5, 1638. 10.1002/adhm.201600048 27109186

[27] S. Chung , H. Lee , H.‐S. Kim , M.‐G. Kim , L. P. Lee , J. Y. Lee , Nanoscale 2016, 8, 14213. 27389611 10.1039/c6nr01956k

[28] E. Roche , Sci. Transl. Med. 2020, 12, eabd3082.

[29] E. Ruel‐Gariépy , J. C. Leroux , Eur. J. Pharm. Biopharm. 58, 409. 15296964 10.1016/j.ejpb.2004.03.019

[30] S. S. Liow , Q. Dou , D. Kai , A. A. Karim , K. Zhang , F. Xu , X. J. Loh , ACS Biomater. Sci. Eng. 2016, 2, 295. 33429534 10.1021/acsbiomaterials.5b00515

[31] S. Lanzalaco , E. Armelin , Gels 2017, 3, 36. 30920531 10.3390/gels3040036 PMC6318659

[32] D. Neradovic , W. L. J. Hinrichs , d. B. J. J. Kettenes‐van , N. C. F. van , W. E. Hennink , Proc. Int. Symp. Control. Release Bioact. Mater. 2000, 27th, 612.

[33] S. Kim , K. E. Healy , Biomacromolecules 2003, 4, 1214. 12959586 10.1021/bm0340467

[34] H. Tekin , J. G. Sanchez , T. Tsinman , R. Langer , A. Khademhosseini , AIChE J. 2011, 57, 3249. 23105146 10.1002/aic.12801 PMC3480343

[35] B. H. Lee , B. Vernon , Polym. Int. 2005, 54, 418.

[36] J. F. Pollock , K. E. Healy , Acta Biomater. 2010, 6, 1307. 19941981 10.1016/j.actbio.2009.11.027

[37] N. Bayat , Y. Zhang , P. Falabella , R. Menefee , J. J. Whalen , M. S. Humayun , M. E. Thompson , Sci. Transl. Med. 2017, 9, eaan3879. 29212712 10.1126/scitranslmed.aan3879

[38] M. Lang , Int. J. Nanomed. 2012, 7, 4893. 10.2147/IJN.S32645 PMC3446841 23028218

[39] A. Fathi , S. M. Mithieux , H. Wei , W. Chrzanowski , P. Valtchev , A. S. Weiss , F. Dehghani , Biomaterials 2014, 35, 5425. 24731705 10.1016/j.biomaterials.2014.03.026 PMC4419780

[40] S.‐K. Hu , T. K. Low , A. Goldstein , Mol. Cell. Biochem. 1981, 41, 49. 7329413 10.1007/BF00225296

[41] S.‐H. Yoon , U. W. Rah , S. S. Sheen , K. H. Cho , Arch. Phys. Med. Rehabil. 2009, 90, 1332. 19651267 10.1016/j.apmr.2009.01.028

[42] W. H. Fang , X. T. Chen , C. T. Vangsness , Arthrosc. Sport. Med. Rehabil. 2021, 3, e1177. 10.1016/j.asmr.2021.01.028 PMC8365196 34430899

[43] O. Kimhi , H. Bianco‐Peled , Langmuir 2002, 18, 8587. 10.1021/la700248s 17444667

[44] H.‐J. Sung , C. Meredith , C. Johnson , Z. S. Galis , Biomaterials 2004, 25, 5735. 15147819 10.1016/j.biomaterials.2004.01.066

[45] M. M. Hasani‐Sadrabadi , P. Sarrion , S. Pouraghaei , Y. Chau , S. Ansari , S. Li , T. Aghaloo , A. Moshaverinia , Sci. Transl. Med. 2020, 12, 6853. 10.1126/scitranslmed.aay6853 32161103

[46] H. Yuk , C. E. Varela , C. S. Nabzdyk , X. Mao , R. F. Padera , E. T. Roche , X. Zhao , Nature 2019, 575, 169. 31666696 10.1038/s41586-019-1710-5

[47] A. Shagan , W. Zhang , M. Mehta , S. Levi , D. S. Kohane , B. Mizrahi , Adv. Funct. Mater. 2020, 30, 1900998.

[48] K. M. R. Nuss , J. A. Auer , A. Boos , B. Von Rechenberg , BMC Musculoskelet. Disord. 2006, 7, 67. 16911787 10.1186/1471-2474-7-67 PMC1578562

[49] R. E. Harrington , T. Guda , B. Lambert , J. Martin , Biomaterials Science, Elsevier, Amsterdam 2020, pp. 1431–1446.

[50] N. P. Tipnis , D. J. Burgess , Int. J. Pharm. 2018, 544, 455. 29274370 10.1016/j.ijpharm.2017.12.003

[51] C.‐S. Chu , A. T. McManus , N. P. Matylevich , C. W. Goodwin , B. A. Pruitt , J. Trauma Acute Care Surg. 2002, 52, 122. 10.1097/00005373-200201000-00021 11791062

[52] P. Abdollahiyan , B. Baradaran , M. de la Guardia , F. Oroojalian , A. Mokhtarzadeh , J. Controlled Release 2020, 328, 514. 10.1016/j.jconrel.2020.09.030 32956710

[53] J. M. Oliveira , L. Carvalho , J. Silva‐Correia , S. Vieira , M. Majchrzak , B. Lukomska , L. Stanaszek , P. Strymecka , I. Malysz‐Cymborska , D. Golubczyk , L. Kalkowski , R. L. Reis , M. Janowski , P. Walczak , npj Regen. Med. 2018, 3, 8. 29644098 10.1038/s41536-018-0046-3 PMC5884770

[54] P. Nezhad‐Mokhtari , M. Ghorbani , L. Roshangar , J. Soleimani Rad , Int. J. Biol. Macromol. 2019, 139, 760. 31400425 10.1016/j.ijbiomac.2019.08.047

[55] Y. Liao , Q. He , F. Zhou , J. Zhang , R. Liang , X. Yao , V. Bunpetch , J. Li , S. Zhang , H. Ouyang , Polym. Rev. 2020, 60, 203.

[56] H. D. N. Tran , K. D. Park , Y. C. Ching , C. Huynh , D. H. Nguyen , J. Ind. Eng. Chem. 2020, 89, 58.

[57] B. Ghosh , M. D. Kirtania , Plant and Algal Hydrogels for Drug Delivery and Regenerative Medicine, Elsevier, Amsterdam 2021, pp. 535–568.

[58] J. M. Blonder , L. Baird , J. C. Fulfs , G. J. Rosenthal , Life Sci. 1999, 65, PL261. 10576602 10.1016/s0024-3205(99)00495-6

[59] J. Shi , L. Yu , J. Ding , Acta Biomater. 2021, 128, 42. 33857694 10.1016/j.actbio.2021.04.009

[60] J. L. Guo , Y. S. Kim , V. Y. Xie , B. T. Smith , E. Watson , J. Lam , H. A. Pearce , P. S. Engel , A. G. Mikos , Sci. Adv. 2019, 5, eaaw7396. 31183408 10.1126/sciadv.aaw7396 PMC6551165

[61] E. T. Pashuck , M. M. Stevens , Sci. Transl. Med. 2012, 4. 10.1126/scitranslmed.3002717 23152328

[62] Y. S. Zhang , A. Khademhosseini , Advances in Engineering Hydrogels, Vol. 356, American Association for the Advancement of Science, Washington, DC, 2017. 10.1126/science.aaf3627 PMC5841082 28473537

[63] V. Pertici , C. Pin‐Barre , C. Rivera , C. Pellegrino , J. Laurin , D. Gigmes , T. Trimaille , Biomacromolecules 2019, 20, 149. 30376309 10.1021/acs.biomac.8b01242

[64] E. Orava , J. Korventausta , M. Rosenberg , M. Jokinen , A. Rosling , Polym. Degrad. Stab. 2007, 92, 14.

[65] S. Flohé , E. Dominguez Fernández , M. Ackermann , T. Hirsch , J. Börgermann , F. U. Schade , Cytokine 1999, 11, 796. 10525319 10.1006/cyto.1998.0490

[66] D. Pappalardo , T. Mathisen , A. Finne‐Wistrand , Biomacromolecules 2019, 20, 1465. 30855137 10.1021/acs.biomac.9b00159

[67] A. Li , B. L. Dearman , K. E. Crompton , T. G. Moore , J. E. Greenwood , J. Burn Care Res. 2009, 30, 717. 19506497 10.1097/BCR.0b013e3181abffca

[68] P. A. Cheshire , M. R. Herson , H. Cleland , S. Akbarzadeh , Burns 2016, 42, 1088. 27222383 10.1016/j.burns.2016.01.028

[69] Y. Wang , S. M. Mithieux , Y. Kong , X. Wang , C. Chong , A. Fathi , F. Dehghani , E. Panas , J. Kemnitzer , R. Daniels , R. M. Kimble , P. K. Maitz , Z. Li , A. S. Weiss , Adv. Healthcare Mater. 2015, 4, 577. 10.1002/adhm.201400571 25469903

[70] H. Wu , S. Han , B. Wu , X. Du , Z. Sheng , J. Lin , X. Chen , K. Zhao , V. Bunpetch , Y. Chen , M. Zeng , E. V. Alakpa , Y. Ma , X. Lei , J. Huang , X. Zou , H. Ouyang , Appl. Mater. Today 2019, 16, 169.

[71] P. Trueba , C. Navarro , J. A. Rodríguez‐Ortiz , A. M. Beltrán , F. J. García‐García , Y. Torres , Surf. Coatings Technol. 2021, 408, 126796.

[72] D. Stankovic , M. Labudovic‐Borovic , R. Radosavljevic , M. Marinkovic , E. R. Isenovic , J. Stomatol. Oral Maxillofac. Surg. 2018, 119, 446. 29747053 10.1016/j.jormas.2018.04.015

