# Supplementary material for: Thermoresponsive and Injectable Hydrogel for Tissue Agnostic Regeneration
Source: Adv Healthc Mater. 2022 Oct 17;11(23):2201714. doi: 10.1002/adhm.202201714 (PMC11468498; doi:10.1002/adhm.202201714)
Supplement: Supplementary file 1 — Supporting Information [file ADHM-11-2201714-s002.pdf]

ADVANCED HEALTHCARE MATERIALS

Supporting Information

for *Adv. Healthcare Mater.*, DOI 10.1002/adhm.202201714

Thermoresponsive and Injectable Hydrogel for Tissue Agnostic Regeneration

Dax Calder, Ali Fathi, Farshad Oveissi, Simin Maleknia, Terence Abrams, Yiwei Wang, Joanneke Maitz, Kevin Hung-Yueh Tsai, Peter Maitz, Wojtek Chrzanowski, Ivan Canoy, Vivek Ashoka Menon, Kenneth Lee, Benjamin J. Ahern, Natasha E. Lean, Dina M. Silva, Paul M. Young, Daniela Traini, Hui Xin Ong, Rasoul Seyed Mahmoud, Hossein Montazerian, Ali Khademhosseini and Fariba Dehghani**

Thermoresponsive and Injectable Hydrogel for Tissue Agnostic Regeneration

Supporting Information

Dax Calder[1,2,3]†, Ali Fathi[1,4]†*, Farshad Oveissi[1], Simin Maleknia[4], Terence Abrams[4], Yiwei Wang[5], Joanneke Maitz[5], Kevin Hung- Yueh Tsai[5], Peter Maitz[5], Wojtek Chrzanowski[2,3], Ivan Canoy[6], Vivek Ashoka[6] Menon, Kenneth Lee[6,7], Benjamin J. Ahern[8], Natasha E. Lean[8], Dina M. Silva[9,10], Paul M. Young[9,10], Daniela Traini[9,10], Hui Xin Ong[9,10], Rasoul Seyed Mahmoud[11], Hossein Montazerian[11,12,13], Ali Khademhosseini[11], Fariba Dehghani[1,]*

PNPHO chemical composition, molecular weight, polydispersity and Thermal characterization

PNPHO is an abbreviation for Poly(N-isopropylacrylamide-co-(N-acryloxysuccinimide)-*co*-(Polylactide/-Hydroxy methacrylate)-co-(Oligo (ethylene glycol). Monomers and macromonomers were reacted in Dimethyl formamide (DMF) under a nitrogen blanket at 70 ºC. By assessing the fraction of unreacted monomers and the initiator in the reactant solution at different time points, it was concluded the reaction was complete after 18 hours. The resulting polymer solution in dimethyl formamide was purified in pre-warmed water for injection at 30 ºC as an anti-solvent. The use of organic solvents, e.g., diethyl ether as an anti-solvent was intentionally avoided to reduce the amount of organic solvent residues in the final product as well as ensure production scalability by mitigating the need for costly and flammable solvents. In addition, water for injection was used, as opposed to distilled water, for the purification of PNPHO polymer to control the endotoxin levels in the final product.

The impact of purification in water in relation to the SEC chromatogram of the polymer and the polydispersity of PNPHO are shown in Figure. S1A. The polydispersity of the polymer solution in DMF was reduced by nearly 2-fold after purification in water from ~3.5 to 1.7 (Mw/Mn). After purification, the chemical composition of PNPHO polymer was assessed via ^{1}H-NMR; the associated characteristic peaks of the polymer to calculate the stoichiometry of its composition is shown in Figure. S1B. The final molar concentrations of building block of PNPHO polymer were calculated based on the integration of the

characteristic peaks; NIPAAm molar composition is equal to the integration of (b), NAS is equal to (e)/4, PLA/HEMA is equal to (h)/lactate number (4 to 6, calculated based on ^{1}H-NMR of PLA/HEMA) and OEGMA is equal to (m)/3. In the current formulation of TX140, PNPHO chemical composition includes 81 mol% NIPAAm, 7 mol% PLA/HEMA, 7 mol% NAS and 5 mol% OEGMA. SEC method showed that PNPHO weight average molecular weight (Mw) is 23 +/- 2 kDa, number average molecular weight (Mn) 16 +/- 2 kDa. The production process of PNPHO polymer is validated in accordance with ISO13485:2016 requirements in relation to the chemical composition, molecular weight and polydispersity.

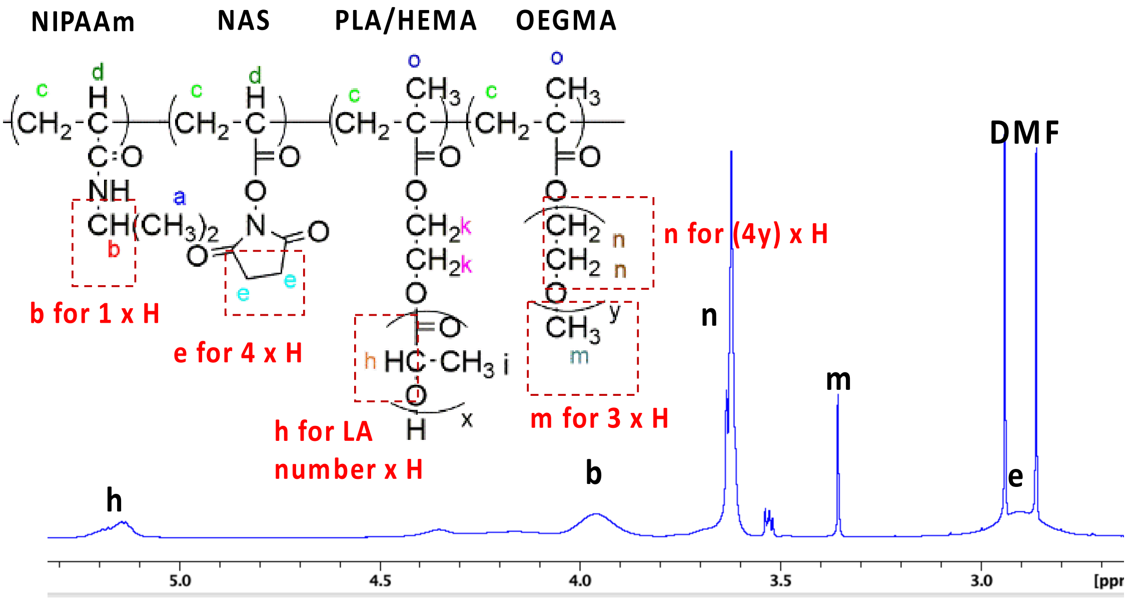

Figure. S1. Chemical analyses of PNPHO polymer. Chemical composition of PNPHO polymer, 1H-NMR characteristic peaks associated with each building block.

Thermal stability and degradation of PNPHO were assessed to confirm the compatibility of the polymer and resulting final product (TX140) with standard terminal sterilization techniques. The thermal stability and degradation of PNPHO were studied by thermogravimetric analysis (TGA) under nitrogen atmosphere, and the thermogravimetric curve and its derivative are presented in Figure. S2. The mass loss plot showed three main processes occurring within the tested temperature range evidenced by the first derivative curve. A dehydration process was observed between 50-100 ºC, resulting from the loss of sample moisture. The calculated water content was 1%. Besides dehydration, two degradative processes were observed, attributed to the two main components of the PNPHO. The first

degradation was at 287ºC, followed by a second degradation step with the largest weight loss, with a maximum at 401ºC. The calculated residue at 600ºC was 8% of the initial weight.

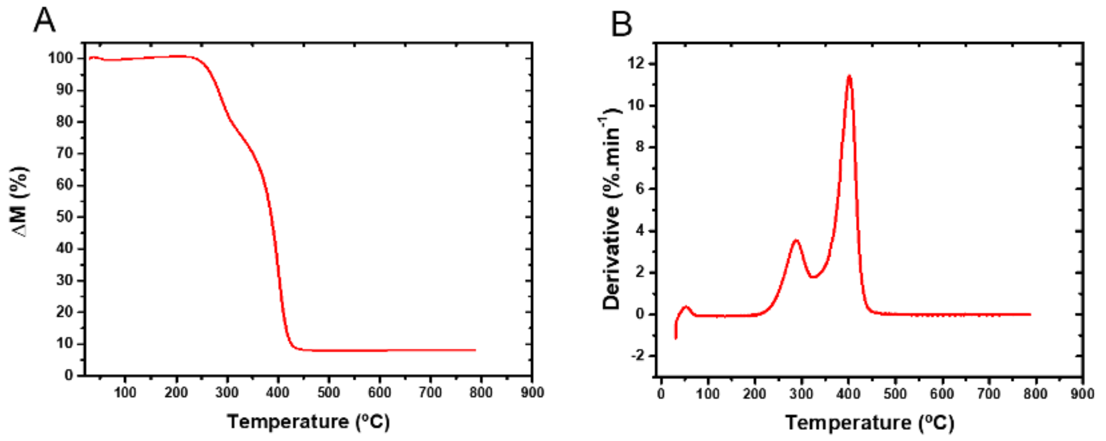

Figure. S2. Thermogravimetric analysis of PNPHO (A) TGA thermal curve of PNPHO recorded at 10°C.min^{-1}, under a nitrogen purge. (B) First derivative of the weight loss curve for PNPHO.

Clinical injectability of PNPHO polymer solutions

PNPHO concentration has a determining impact on the viscosity of the final product as preliminary studies confirmed that the addition of Thymosin ß4 has no significant impact on the viscoelasticity and flowability of the final product. In the context of clinical usability, the flowability of the polymer solution through 22G needle was identified as a critical requirement to ensure that the final product is amenable to both superficial- and deep-tissue administration [41,42]. Therefore, PNPHO solutions with different polymer concentrations were formed and injectability assessment was carried out by three independent clinicians to determine the optimized PNPHO concentration. PNPHO polymer solutions with a polymer content of less than 70 mg.ml^{-1} fails to form cohesive hydrogels upon the increase of temperature to 37 ºC and thus 70 mg.ml^{-1} was used as the lower limit for this investigation. The polymer content was further increased by 1.5X to 4X with 0.5X intervals. Accordingly, a range of PNPHO solutions in phosphate buffer saline (PBS) with polymer concentration in the range of 70 mg.ml^{-1} to 280 mg.ml^{-1} were formulated. The results from the extrudability study are presented in Table S1. The results showed that PNPHO solution with the concentration of 140 mg ml^{-1} can be extruded conveniently through a 22G needle by using a standard polypropylene syringe.

Table S1. Injectability assessment of PNPHO solutions. Extrudability of different PNPHO polymer solution through different needle gauge size
size

PNPHO Concentration (mg. ml-1)	Needle gauge size (Internal Diameter, mm)						
	18G (0.838 mm)	19G (0.686 mm)	20G (0.603 mm)	21G (0.514 mm)	22G (0.413 mm)	23G (0.337 mm)	25G (0.260 mm)
**70**							+++
**105**						+++	
**140**					+++		
**175**			+++				
**210**			+++				
**245**		+++					
**280**	+++						

Rheological characterization and injectability quantification

The required forces to extrude PNPHO solution with the polymer concentrations of 105 mg.ml^{-1}, 140 mg.ml^{-1}, 175 mg.ml^{-1} and 210 mg.ml^{-1} were measured with Instron. The polymer solutions were loaded into standard polypropylene syringe syringes and fitted with a 22G needle. The forcing pressure was measured to be able to fully extrude 1ml of the solutions within 12 seconds (selected as a clinically relevant injection duration). The results showed that the injection force of solutions with PNPHO concentrations of 105 mg.ml^{-1} and 140 mg.ml^{-1} can be fully extruded through a 22G needle with injection force less than 5N which was significantly lower than solutions formed with 175 mg.ml^{-1} and 210 mg.ml^{-1}. Viscosity-shear rate characteristics of PNPHO solutions formed with PNPHO concentrations at 37 ºC is shown in Figure. S3A. The decreasing trend of viscosity with shear rate is indicative of shear-thinning behavior. Shear stress increases linearly with strain in Figure. S3B. The results showed the elastic region, followed by an abrupt decrease in stress, representing network rupture at the yield point. As expected, the shear stress at the yield point increases with polymer concentration. These results further endorse the findings from clinical injectability assessments and showed that PNPHO solution with 140 mg.ml^{-1} polymer

concentration exhibits the optimum characteristics to ensure easy and effective administration of the product. In addition, the adhesion strength of solutions with different polymer concentration were evaluated and shown in Figure. S3C. To this end, a tack test was performed using a rheometer (Anton Paar, MCR 302, USA). A 25 mm diameter torque measurement system was used to measure hydrogel tackiness. After injecting hydrogels at a low temperature (4 °C), the temperature was raised to 37 °C, and samples were left for thermal stabilization for 10 min. Next, a vertical motion profile at 0.5 mm/s displacement rate was implemented to the samples from the initial gap size of 1 mm. The result showed that the solution formed with 140 mg.ml^{-1} displayed adhesion force of 72 +/- 10 mN. The magnitude of adhesion force increased with the concentration of the polymer solutions due to the increase in the density of interfacial interactions as well as cohesion in hydrogels.

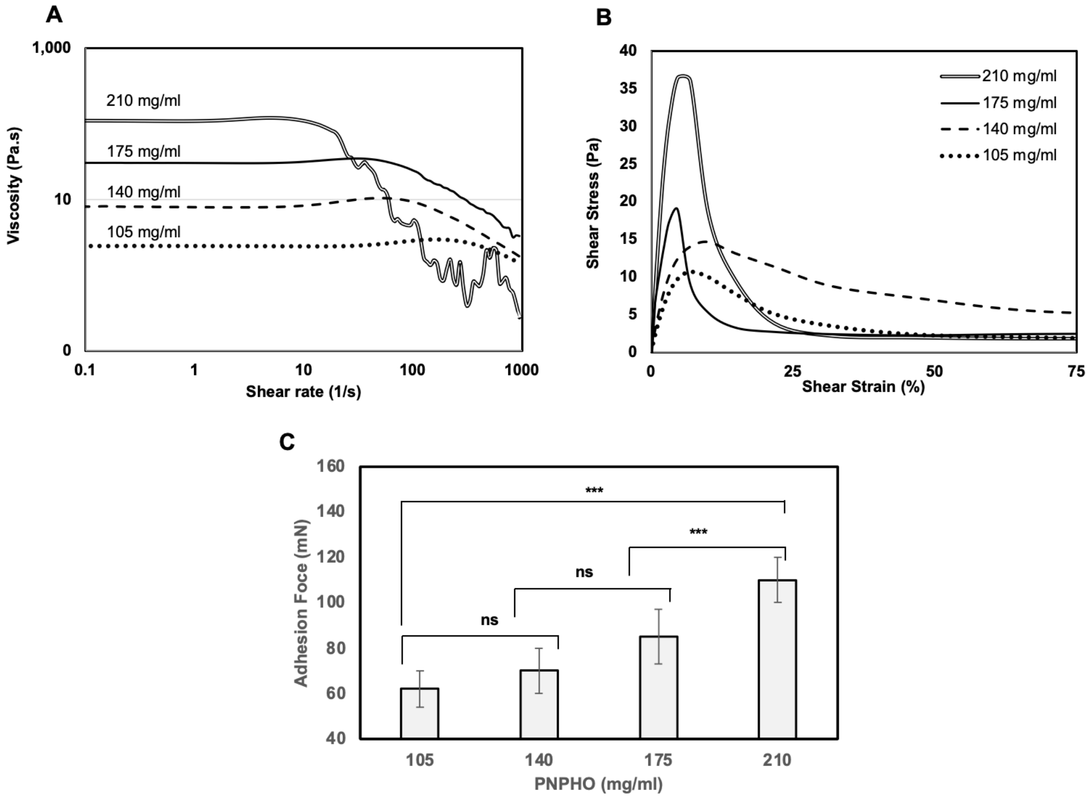

Figure. S3. Rheological and adhesion characterization of prepolymer solutions. (A) Viscosity-shear rate characteristics of aqueous solutions at 37 ºC. The decreasing trend of viscosity with shear rate is indicative of shear-thinning behavior. (B) Variation of shear stress versus shear strain in oscillatory rheological tests. Shear stress increases linearly with strain in the elastic region, followed by an abrupt decrease in stress, representing network rupture at the yield point. Shear stress at yield point increases with polymer concentration. (C) Analysis

of adhesion force measured through a tensile adhesion setup (tack test) following a gelation process at 37 ºC.

LCST via 1H-NMR and Thymosin-β4 concentration optimization

The LCST of PNPHO polymer solution conjugated with different amounts of Thymosin-ß4 is driven by the chemical composition of PNPHO, Thymosin-ß4 and the ratio between hydrophilic to hydrophobic groups in the molecular structure of the conjugated system. In accordance with the method developed by Kimhi and Bianco-Peled [43], the coil to globe transition behavior of the temperature responsive matrices at their LCST or higher, immobilizes hydrogen ions, causing a reduction in the integration of characteristic peaks (b), (n), (m) or (h), which reflected in the increase of D_2O/(b), D_2O/(m), D_2O/(n), D_2O/(h). Accordingly, D_2O/(b), D_2O/(m), D_2O/(n) and D_2O/(h) at different temperatures were plotted to identify the most accurate method to identify the LCST measurement. D_2O/(m) was the best representation of the acquired due to the baseline and unsymmetric nature of the peaks at (n), (b) and (h). Upon the increase of temperature, the ^{1}H-NMR signal of hydrogel drops, thus increasing the value of D_2O/(m); accordingly, the LCST of the system was identified from the intersection of a line drawn through the baseline and the leading edge of the curve.

Thymosin-β4 concertation was varied in the range of 0 to 60 mg.ml^{-1} and the LCSTs of the resulting solutions comprising of 140 mg.ml^{-1} of PNPHO (fixed amount) were identified by acquiring ^{1}H-NMR spectra at different temperatures. D2O/(m) measurements at different temperatures ranged from 16 ºC to 26 ºC for PNPHO-*co*- Thymosin-β4 solutions formulated with 140 mg.ml^{-1} of PNPHO and different concentrations of Thymosin-β4 are depicted in Figure. S4. Results showed that the LCST of PNPHO polymer is 24 ºC. However, crosslinking of PNPHO polymer with 20 mg.ml^{-1} and 30 mg.ml^{-1} of Thymosin-β4 decreases the LCST from 24 ºC to 22 ºC and 21 ºC respectively. The LCST of PNPHO-*co*–Thymosin-β4 solution plateaus with 30 mg.ml^{-1} of Thymosin-β4 at 21 ºC; a further increase of Thymosin-β4 concentration to 40 mg.ml^{-1}, 50 mg.ml^{-1} and 60 mg.ml^{-1} had no impact on the LCST of the solution. To this end, it was concluded that 30 mg.ml^{-1} of Thymosin-β4 was ideal to crosslink 140 mg.ml^{-1}of PNPHO in TX140.

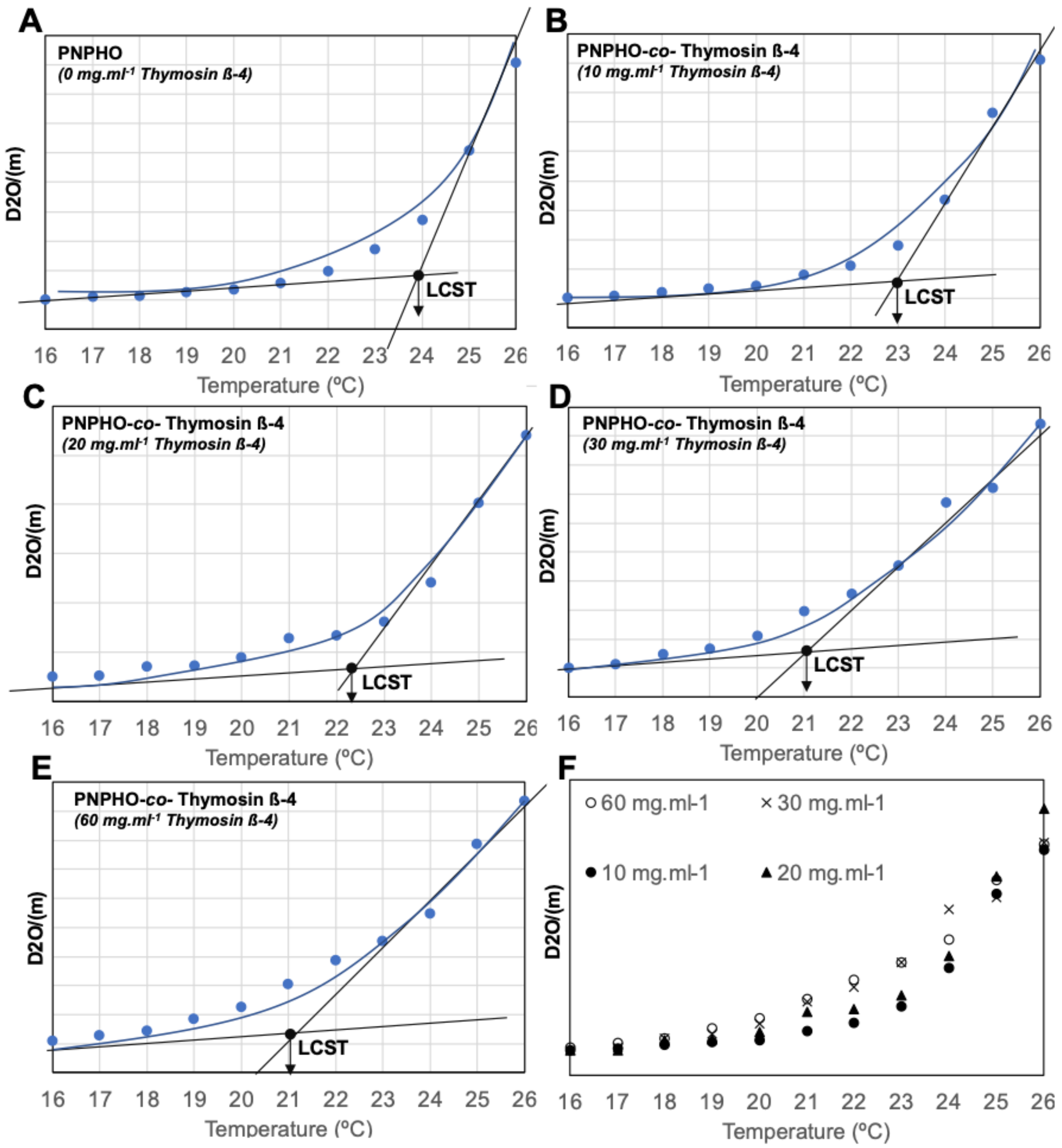

Figure. S4. LCST of PNPHO-co-Thymosin-ß4 formed with different Thymosin-ß4 concentration. D2O/(m) at different temperature for solutions with 140 mg.ml^{-1} of PNPHPO and **(A)** 0 mg/ml^{-1} of Thymosin ß-4; **(B)** 10 mg/ml^{-1} of Thymosin ß-4; **(C)** 20 mg/ml^{-1} of Thymosin ß-4; **(D)** 30 mg/ml^{-1} of Thymosin ß-4; **(E)** 60 mg/ml^{-1} of Thymosin ß-4 and **(F)** D2O/(m) of all solutions at different temperature ranges.

Biological effects of undiluted PNPHO, TX140, and Thymosin-β4 extracts in healthy-derived bronchial cells

The biological effects of PNPHO, TX140 and Thymosin-β4 on Beas-2B cells were evaluated by cell viability, IL-6 levels, and Live/Dead staining after the exposure to the extracts diffused through the membrane of a cell culture insert for 24h. No cytotoxic effects were observed on Beas-2B cells for PNPHO and TX140 hydrogels, respectively (Figure. S6A). The pro-inflammatory effects of the extracts showed that PNPHO and TX140 did not induce any IL-6 increase on the basal levels whereas Thymosin-β4 peptide significantly decreased the IL-6 basal levels (Figure. S6B). An additional LIVE/DEAD viability assay was conducted after exposure to the extracts, by staining the live cells with calcein-AM (green) and dead cells with EthD-1 (red). Nuclei were stained with Hoechst and are depicted in blue. The representative microphotographs in Figure. S6-C show similar live (green) cell densities per optical field across test samples and between the extracts and control (media only). These results further support that both hydrogel formulations are not toxic to healthy-derived bronchial cells.

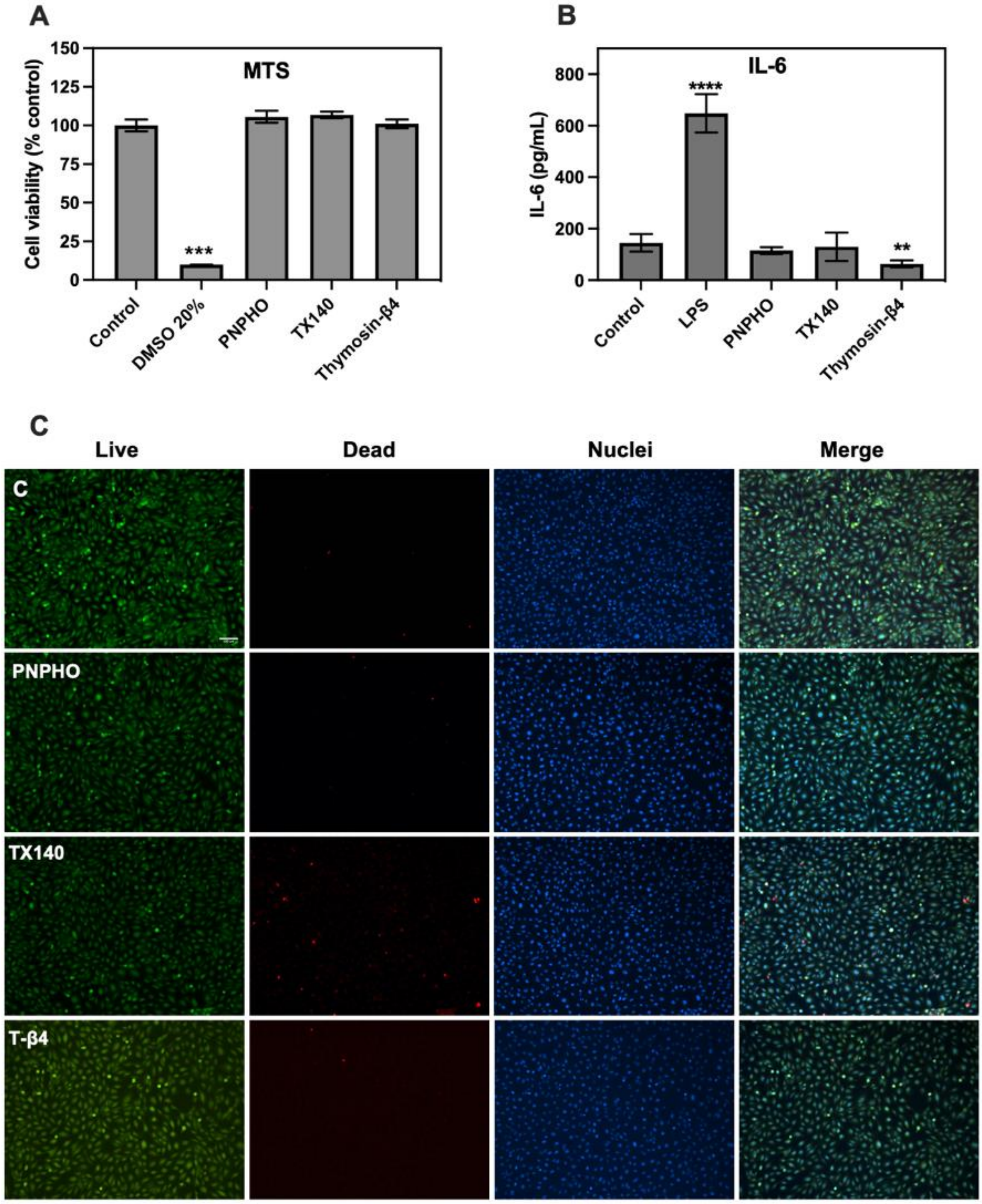

Figure. S5. **Biological effects of the undiluted extracts.** (A) Cell viability expressed as percentage of the negative control (media only) and (B) IL-6 levels of healthy-derived bronchial cells (Beas-2B) after exposure to the extracts of PNPHO, TX140 and Thymosin-$\boldsymbol{\beta}$4 for 24h. Statistical differences between control and treatments were evaluated by an unpaired t-test using GraphPad Prism software (**p<0.01; ***p<0.001; ****p<0.0001). (C) Representative images (10x magnification) of Beas-2B cells after exposure to the extracts for

24h and stained with Calcein-AM (green), EthD-1 (red), Hoechst (blue) and overlay of the 3 channels. Scale bar – 100 μm.

***Ex vivo* test for assessing injectability, gelation, and adhesive property of TX140**

A medial femoral condyle osteochondral defect in cadaveric sheep hindlimbs was formed to investigate the usability, injectability, gelation and adhesive properties of TX140 hydrogel in a simulated physiological condition. A protocol was generated with predefined acceptance criteria for TX140 injectability, gelation and adhesion as listed in Table S2. The tissues were placed in a water bath to maintain the temperature at ~37 ºC to simulate a live animal model. Six medial femoral condyles (MFC) of cadaveric sheep hindlimbs were used to assess the *ex vivo* injectability, gelation and adhesivity of TX140. All limbs were disarticulated from the body at the coxofemoral joint and placed in a water bath at 37 ºC for 1 hour before further testing. A standard arthrotomy to access the MFC of the femur was made on each limb whilst inflexion. A 6 mm diameter and 6 mm deep defect was drilled through the central weight-bearing aspect of the MFC and flushed with saline at 40 ºC. Six (6) defects were filled with TX140 and allowed for temperature induced gelation. Once gelation was confirmed, the stifle was taken through a standard range of motion 20 times and the filled defect assess for any movement of the fabricated hydrogel system. If no movement was detected, then the stifle went through another extra 20 range of motions and the evaluation continued for up to 100 range of motions in total (see Table S2).

Table S2. TX140 usability assessment in a contained site under dynamic motion; Injectability, gelation and adhesivity analyses of TX140 in a chondral defect mode.

**Element**	**Acceptance Criteria**	**Results***	**Pass/Fail**
**Injectability**	Complete extrusion of TX140 content through 22G needle into condyle osteochondral defect	6/6 injectable	**PASS**
**Gelation**	Hydrogel formation of TX140 solution post extrusion through 22G needle at the condyle osteochondral defect site at 37°C	6/6 forms hydrogel	**PASS**
**Adhesivity**	Presence/ adhesion of the gelled TX140 at the condyle osteochondral defect site (6 x 6 cm) after 100 range of motion	6/6 adhesion	**PASS**

*: use +/- for confirmation of injectability, gelation and adhesivity

TGA characterization of TX140

DSC curves were obtained for the range of temperatures below the degradation steps (< 200ºC) for 3 heating/cooling cycles, depicted in Figure. S5A and Figure. S5B. A broad endothermic peak was observed in the first heating cycle due to dehydration. In the second and third heating cycles a glass transition process between 50- 100 ºC (T_g=61 ºC) was observed. No further processes were observed after multiple heating-cooling cycles. The thermal stability of TX140 was evaluated between 30 and 800 ºC, showing a dehydration and two degradation steps at 287 ºC and 401 ºC, with a residue of 8%. The DSC curves showed a glass transition, visible only after the first heating cycle, that occurred at 61 ºC.

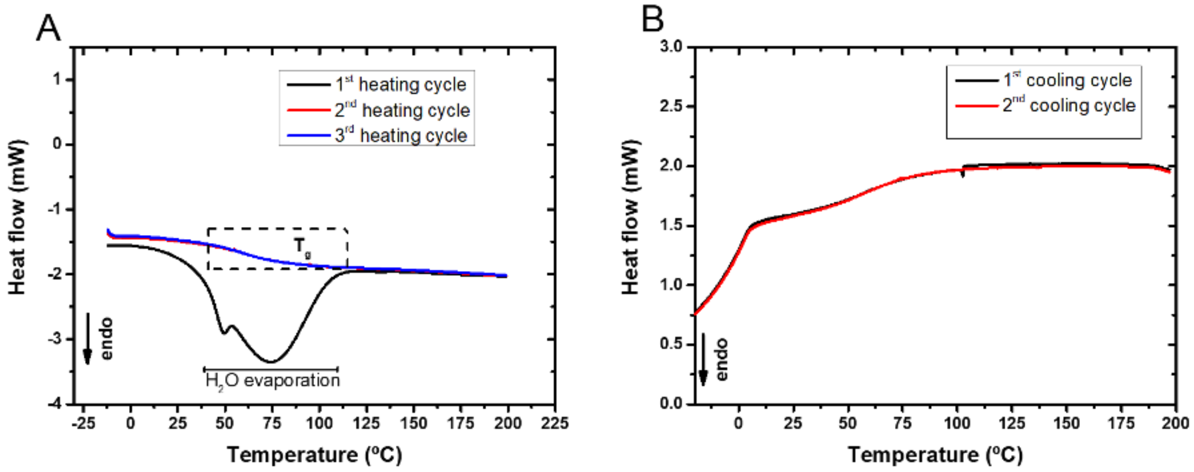

Figure. S6. Thermal stability assessment of TX140. DSC curves under nitrogen atmosphere: **(A)** DSC curves for 3 heating cycles and **(B)** DSC curves for 2 cooling cycles.

Gamma irradiation compatibility

Sterilization of TX140 by gamma irradiation at 25 – 40 kGy at the Steritech NSW facility has been demonstrated to comply with relevant sections of ISO 11137-1, ISO 11137-2 and ISO 11137-3 and EN 556-1, and a SAL of 10^{-6} has been established as required per EN 556-1 to designate TX140 as 'sterile' by gamma irradiation. In accordance with ISO11137-Part 2, the compatibility of the TX140 and its constituents on the maximum Gamma dose of 40 kGy was assessed by using a wide range of chemical analyses, benchtop testing as well as live *in vivo* animal studies. The data presented in Table S3 underpins that gamma irradiation even at such a high dose of 40 kGy has no significant impact on the physico-chemical and biological properties of TX140.

Table S3. TX140 compatibility with Gamma radiation for terminal sterilization. Assessments methods to confirm the compatibility of TX140 and its constituents with Gamma irradiation at 25-40 kGy based on predefined acceptance criteria.

Test(s) being Performed	Acceptance Criteria	Results
^{1}H-NMR, chemical composition of PNPHO raw material before and after gamma irradiation	No significant difference between the chemical composition of PNPHO polymers (all four components) between groups, based on One-Way ANOVA test with *p* value of 0.05.	**PASS**<br>The *p* value between the variation of different building blocks before and after Gamma irradiation were as follows; PLA/HEMA; *p=0.13,* NIPAAm; *p*=0.40, OEGMA; *p*=0.83, NAS; *p*=0.19
LC/MS, chemical analyses of TB4 raw material	10/10 identical elution of TB4 in the LC/MS spectra at the relevant elution time for samples before and after gamma irradiation.	**PASS**<br>Identical elution of TB4 for all tested samples before and after Gamma irradiation
TX140 basic performance (extrudability, gelation and swelling ratio).<br><br>Product meet pre-defined acceptance criteria (met for TX140 before	Extrudability of TX140 is confirmed if ER is equal or greater than 0.95 g/ml (ER >=0.95 g/ml). This equates to 95% extrusion of the content from the syringe with confidence level (CL) of 99% and probability of conformance to specification (PCS) of 99%.	PASS<br>Greater than 99% of the product was successfully extruded.
	Gelation of 1 ml of TX140 occurs in 2 minutes or faster (via conduction heat transfer) with confidence level (CL) of 99% and probability of conformance to specification (PCS) of 99%.	PASS<br>Gelation was completed within 30 seconds (via conduction in 37 ºC incubator).

Gamma irradiation) with	TX140 holds water equal or more than 20% of its dry weight post formation of hydrogel in simulated physiological conditions. As such the SR (swelling ratio) equal or more than 1.2 with confidence level (CL) of 99% and probability of conformance to specification (PCS) of 99%.	PASS<br>TX140 product swelling ratio of 2.9 +/- 0.2 (g/g)
^{1}H-NMR/ LCST; chemical analyses of TX140 (PNPHO -*co*-TB4)	No significant difference between the LCST of TR001 before and after gamma irradiation based on One-Way ANOVA test with *p* value of 0.05.	**PASS**<br>No significant difference (*p>0.05)* between the LCST of TX140 before and after gamma irradiation
LC/MS, chemical analyze of TX140 (PNPHO -*co*-TB4)	10/10 identical elution of TB4 and PNPHO in the LC/MS spectra at the relevant time for samples before and after gamma irradiation.	**PASS**<br>Identical elution of TB4 and PNPHO for all tested samples before and after Gamma irradiation
TX140 biological properties	No significant difference between the biological properties of aseptically manufactured and Gamma sterilized TX140 by injecting 2ml of the products subcutaneously in a mice model.	**PASS**<br>No difference between two groups noticed in respect to hematology, internal organs, animal wellbeing, systemic or local inflammation was noticed.

Organic solvent residues

In the synthesis of PNPHO polymer dimethyl formamide (DMF) a USP-Class 2 solvent is used. The maximum allowed residues of DMF per ICH Q3C (R5) is 880 ppm in the final product. In the synthesis of subassembly PLA/HEMA, tetrahydrofuran (THF) and toluene (TOL,) are used; the maximum allowed residues for THF is 720 ppm and for TOL is 890

ppm, in the final product. In addition, for the purification of PLA/HEMA, ethyl acetate (EA) is used, which is USP Class 3; the maximum allowed EA residue is 5000 ppm in the final product. PNPHO concentration in the final product (TX140) is 14 wt/v% and thus the maximum allowed residues of organic solvents in PNPHO were calculated and presented in Table S4. In PNPHO, the maximum allowed residue for DMF is 6,286 ppm, for THF is 5,143 ppm, for Tol is 6,357 and 35,714 ppm for EA. The results showed that DMF residues in PNPHO polymer 110 +/- 45 ppm which is significantly less the maximum allowed in PNPHO (6,286 ppm).

Table S4. The residues of organic solvents in TX140. the amount of DMF, Tol, THF and EA in PNPHO, and the corresponding maximum allowable limit in TX140 based on ICH Q3C (R5) guidelines.

**Organic solvent**	**Maximum in TX140 (ICH Guidelines)**	**Maximum (ppm) level in PNPHO[1]**	**Results (ppm)**	**Pass/ Fail**
Dimethyl formamide (DMF)	880	6,286	Max 150	PASS
Toluene (Tol)	890	6,357	Max 0.2	PASS
Tetrahydrofuran (THF)	720	5,143	Max 0.1	PASS
Ethyl acetate (EA)	5000	35,714	Max 0.3	PASS

1: Maximum in subassembly PNPHO was calculated based on 14 wt/v% concentration of PNPHO in TX140.

Endotoxin level

The endotoxin level in the final product was measured with a kinetic limulus Amebocyte Lysate (LAL) test in accordance with FDA guidance for industry, pyrogen and endotoxin testing, June 2012, USP<85> bacterial endotoxin and Test for Bacterial Endotoxins; British Pharmacopeia, Appendix XIV C. This part of the study was carried out by Eurofins ams Laboratories (New South Wales, Australia). The method validation for TX140 was successfully completed and the standard curve for the measurement was established by using 5 $EU.ml^{-1}$, 0.5 $EU.ml^{-1}$, 0.05 $EU.ml^{-1}$ and 0.005 $EU.ml^{-1}$. The extraction from each TX140 syringe was diluted in 10 ml purified water at 37 ºC for 30 minutes. The result from the study for three consecutive batches of TX140 is summarized in Table S5. The results indicate that

the level of endotoxins in TX140 is <0.100 EU.ml^{-1} for all tested samples which was well within the acceptance criteria.

Table S5. Endotoxin levels in TX140. Endotoxin assessment of TX140 based on predefined acceptance criteria per USP<85>.

**Product**	**Specification per device (20ml)**	**Specification (per ml)**	**Results**	**Pass/ Fail**
TX140 (three batches)	< 24 EU	<1.2 EU.ml^{-1}	<0.100 EU.ml^{-1}	PASS

Investigating the tolerability and acute toxicity of TX140 *in vivo* (Hematology and Biochemistry of Acute Dose Study)

The study to investigate the acute single dose toxicity of TX140 in rats was adapted from the International Organization for Standardization, International Standard ISO 10993-11 Biological evaluation of medical devices Part 11: Tests for systemic toxicity, 2nd edition, 2006 (2). Briefly, on completion of the acclimation period, the study was commenced with administration of the polar and non-polar extract formulations of TX140 (administration was staged between 0.1, 0.5 and 2.0 ml.kg-1 dose levels). The findings were assessed by comparison of outcomes with +Control groups which involve polar and non-polar solvents (with no TX140 extracts).

The hematology parameters such as hemoglobin, red blood cell count, hematocrit, mean cell volume, mean cell hemoglobin concentration, mean cell hemoglobin, reticulocyte counts (% and absolute), white blood cell count, differential white cell count with cell morphology such as Neutrophil, Lymphocyte, Monocyte, Eosinophil, Basophil, and platelet count were investigated. Results in Figure. S7 showed that there is no dose dependent variation in any of the tested hematology parameters and the outcomes were similar ($p>0.05$) among groups including +Control groups (polar and non-polar solvents).

The biochemistry parameters in serum samples such as sodium, potassium, chloride calcium, inorganic phosphorus glucose, urea creatinine, creatinine phosphokinase, total bilirubin, total cholesterol triglycerides, alkaline phosphatase, aspartate aminotransferase, alanine aminotransferase, total proteins, albumin/globulin ratio, Albumin, and Globulin were determined using a Beckman Coulter AU 680 analyzer. The results in Figure. S8 showed that

there is no dose dependent variation in any of the tested biochemistry parameters in serum samples.

It should be noted that there were findings in two rats at the injection site which were attributed to the injection procedure, including a minor scab and swelling at the injection site. This finding was correlated with the presence of an abdominal hernia and the presence of abdominal fat in the subcutaneous space and therefore not related to the dose injections. Also, there were necropsy findings of white deposits in the peritoneal cavities of rats treated with both non-polar vehicle and non-polar TX140 extracts which were attributed to the vehicle treatment (and therefore not related to TX140). There were also findings of red spots on the surface of the lungs of the rats treated with the mid and high dose of the polar and non-polar TX140 extracts and a single finding of white spots on the lungs in one rat treated with a mid-dose of the non-polar TX140 extract. These findings were not associated with any microscopic findings and are considered incidental and unrelated to treatment. Other microscopic findings included minimal alveolar histiocytosis and minimal to moderate lymphohistiocytic interstitial inflammation which was not limited to one group or sex and were considered unrelated to treatment.

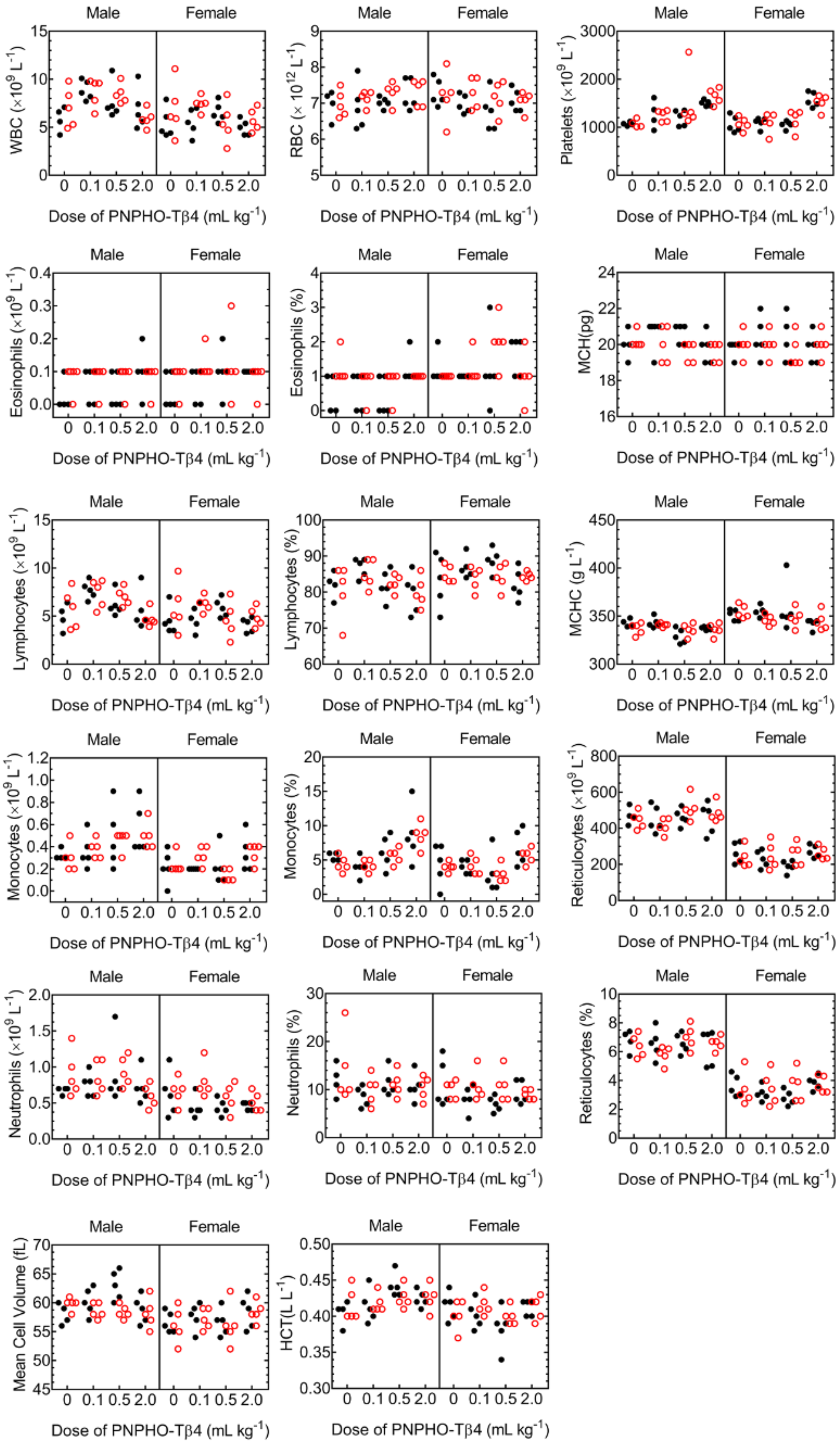

Figure. S7. Hematology results in acute dose study. Polar and non polar extracts are distinguished with closed ● and opened circles ○, respectively. WBC, white blood cells;

RBC, red blood cells; MCH, mean cell hemoglobin; MCHC, mean cell hemoglobin concentration; HCT, hematocrit.

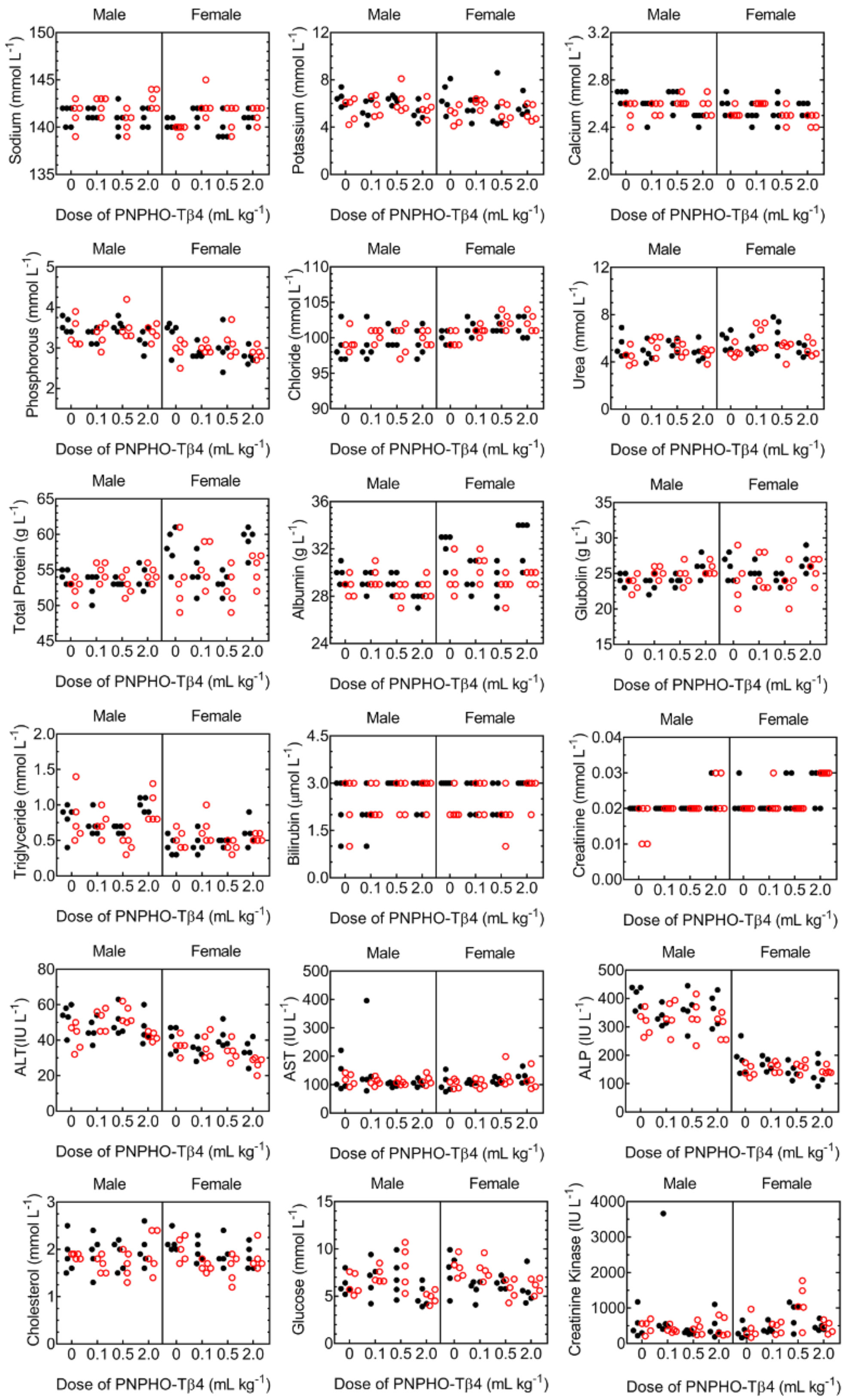

Figure. S8. Biochemistry results in acute dose study. Polar and non-polar extracts are distinguished with closed ● and opened circles ○, respectively. ALP, alkaline phosphatase; AST, aspartate aminotransferase; ALT, alanine aminotransferase.

Long term biocompatibility, internal organ assessments

Each mouse was injected with up to 8% (v/w) of their body weight, distributed over two injection sites (up to 2 ml per mouse). The weight of each mouse was recorded at pre- and post-injection and day 1, day 2 and day 3 and every week following injection (Figure. S9). At time points 1 week, 2 weeks, 4 weeks and 6 weeks post injection, mice were ethically euthanized for collection of tissue biopsies to analyze local response and autopsies performed to analyze systemic toxicology. The results from the macroscopic and microscopic assessment of the sites showed that TX140 samples were fully resorbed within 2-4 weeks post administration. Blood samples were collected by cardiopuncture used for blood cell counting. Serum samples collected by centrifugation of blood were used for measuring serum levels of AST, ALPL creatinine, urea, and albumin by Elisa assays.

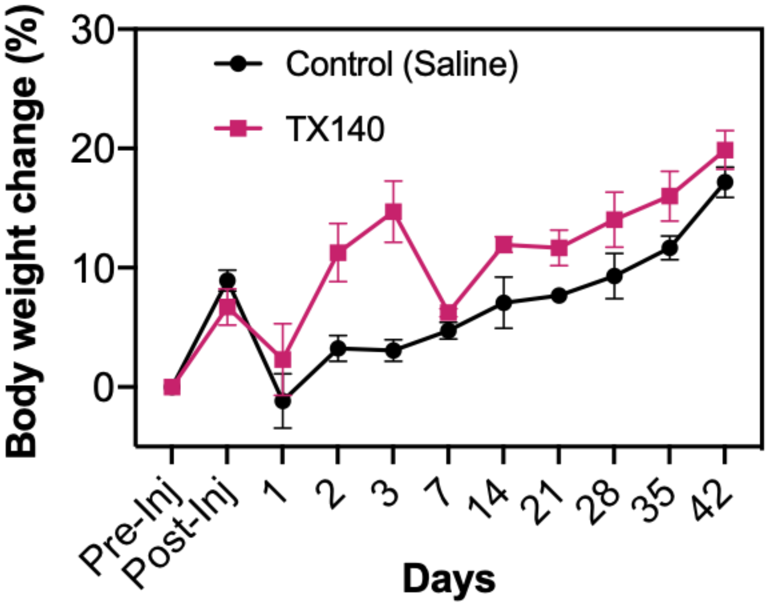

Figure. S9. Animal weight gain. Treated with injected with ~2ml TX140 or 2ml saline after 6 weeks (n=24 animals in total, 6 sacrificed on day 14, 6 animals sacrificed on day 28 and 12 sacrificed at termination on day 42).

Internal organs including liver, lung, kidney and spleen were harvested, weighed, and histologically analyzed by two independent assessors at the Department of Pathology of Concord Repatriation General Hospital (New South Wales Australia). The results from the histopathological analyses of internal organs are summarized in Table S6. Focal and mild extramedullary hematopoiesis was found in both controls and TX140 injection study groups. Extramedullary hematopoiesis is the production of blood cells outside of the bone marrow and is normally observed in physiological processes in embryonic stages. As the extramedullary hematopoiesis was observed in both control (saline injection) and TX injection study groups, it is suggestive of a mouse colony related anomaly and not related to the TX140 injections. No granulomas or excessive hepatic inflammation was observed. The findings together with liver weight and AST and ALPL results, suggests no acute liver failure or pathology was present in treatment with TX140. No abnormality was observed in kidney histopathological analysis in control and TX140 treated animals. Granulomatous inflammation of the lungs was noticed which is defined by the presence of large groups of histiocytes in tissue. No abnormality was observed in the spleen histology of control and TX140.

Table S6. Histological assessments of internal organs. Summary of findings in relation to the histopathological assessment of internal organs by two blinded assessors.

**Animal ID**	**Group**	**Time Point**	**Assessor 1**				**Assessor 2**			
			**Liver[1]**	**Lung[2]**	**Kidney[3]**	**Spleen[3]**	**Liver[1]**	**Lung[2]**	**Kidney[3]**	**Spleen[3]**
Con-1	Saline	1 Week	NAD	0	NAD	NAD	0	0	NAD	NAD
Con-2	Saline	1 Week	NAD	0	NAD	NAD	0	0	NAD	NAD
Con-3	Saline	1 Week	F-EMH	0	NAD	NAD	F-EMH	0	NAD	NAD
Con-4	Saline	2 Week	NAD	0	NAD	NAD	0	0	NAD	NAD
Con-5	Saline	2 Week	NAD	0	NAD	NAD	0	0	NAD	NAD
Con-6	Saline	2 Week	NAD	0	NAD	NAD	0	0	NAD	NAD
Con-7	Saline	4 Week	NAD	0	NAD	NAD		0	NAD	NAD
Con-8	Saline	4 Week	F-EMH	0	NAD	NAD	F-EMH	0	NAD	NAD

Animal ID	Group	Time Point	Assessor 1				Assessor 2			
			Liver[1]	Lung[2]	Kidney[3]	Spleen[3]	Liver[1]	Lung[2]	Kidney[3]	Spleen[3]
Con-9	Saline	4 Week	NAD	0	NAD	NAD	0	0	NAD	NAD
Con-10	Saline	6 Week	F-EMH	0	NAD	NAD	F-EMH	0	NAD	NAD
Con-11	Saline	6 Week	NAD	0	NAD	NAD	0	0	NAD	NAD
Con-12	Saline	6 Week	NAD	0	NAD	NAD	0	0	NAD	NAD
TX140-13	TX140	1 Week	F-EMH	0	NAD	NAD	F-EMH	0	NAD	NAD
TX140-14	TX140	1 Week	NAD	0	NAD	NAD	0	0	NAD	NAD
TX140-15	TX140	1 Week	NAD	0	NAD	NAD	0	0	NAD	NAD
TX140-16	TX140	2 Week	NAD	0	NAD	NAD	F-EMH	0	NAD	NAD
TX140-17	TX140	2 Week	F-MEH	0	NAD	NAD	0	0	NAD	NAD
TX140-18	TX140	2 Week	NAD	0	NAD	NAD	0	0	NAD	NAD
TX140-19	TX140	4 Week	NAD	1	NAD	NAD	0	1	NAD	NAD
TX140-20	TX140	4 Week	F-EMH	1	NAD	NAD	F-EMH	1	NAD	NAD
TX140-21	TX140	4 Week	NAD	1	NAD	NAD	0	1	NAD	NAD
TX140-22	TX140	6 Week	NAD	2	NAD	NAD	0	2	NAD	NAD
TX140-23	TX140	6 Week	NAD	2	NAD	NAD	0	2	NAD	NAD
TX140-24	TX140	6 Week	NAD	2	NAD	NAD	0	2	NAD	NAD

1: Liver score; NAD: No abnormality detected, and F-EMH; focal extramedullary hematopoiesis

2: Lung score; 0: no granulomatous inflammation, 1: focal/mild inflammation, 2: moderate inflammation and 3: severe inflammation.
3: Kidney and spleen score; NAD: No abnormality detected

Soft-tissue repair and angio-conductive properties of TX140

A full thickness dermal mice model was used to assess the capability of TX140 to support soft tissue healing, cell infiltration, neo-vascularization and thus integration with soft tissue. The dorsum of each mouse (n=27) was shaved and two adjacent and identical full-thickness skin grafts of 1 cm^2 were surgically excised. The full-thickness wounds were covered with either TX140 or a collagen membrane (Integra, +C) followed by full-thickness skin grafts from the opposite site superimposed onto either TX140 or +C. Skin grafts were sutured in place. At different time points, skin survival rate, blood vessel ingrowth, cell infiltration and angiogenesis within Integra and TX140 were analyzed and assessed based on pre-defined acceptance criteria. Summary of assessments, the pre-defined acceptance criteria and the outcomes are presented in Table S7.

Table S7. Soft tissue healing application of TX140. Summary of performed tests, acceptance criteria, acquired results and the associated protocol pass/fail conclusion.

**Test(s) being Performed**	**Acceptance Criteria**	**Results**	**Protocol Pass/Fail**
Skin graft survival rate	The survival rate of skin grafts in TX140 treated animals is equal or greater than that of in Integra treated site in animals alive at different time points	100% survival for PNPHO-Tβ4 treated site;<br>85% survival rate for Integra treated site	**PASS**[1]
Blood vessel ingrowth	Fluorescent illumination of the skin graft site treated with PNPHO-Tβ4 is equal (null hypothesis accepted with *p<0.05)* or greater than (significant difference with	Significantly higher (p<0.05) radiant efficiency of TX140 treated sites compared to Integra after one week post-surgery<br>Significantly higher (p<0.01) radiant efficiency of TX140 treated	**PASS**

	p<0.05*)* that of treated with Integra (with one-way ANOVA testing) at different time points after 24 hours of injection of AngioSense750 EX in vivo blood pool fluorescent	sites compared to Integra after two weeks post-surgery<br>Equal radiant efficiency of TX140 and Integra treated sites after 4 weeks post-operation	
Integration with soft tissue	Semi-quantitative measure/ multiple interlinked measurements	Apparent less fibrous tissue formation around TX140 biomaterial at the dermal site compared with Integra treated sites<br>Apparent more cell infiltration throughout the structure of TX140 compared with Integra treated sites<br>Apparent more blood vessel ingrowth within the structure of TX140 in comparison with Integra treated site<br>Apparent formation of more (thicker) dermal connective tissue within TX140 in comparison with Integra treated sites	**PASS**

[1]**PASS** – All individual test results HAVE MET the pre-defined acceptance criteria per the corresponding protocol (acceptance criteria include test results, failure mode acceptance, confidence intervals, upper and lower limits, etc., as appropriate.

Hard-tissue repair with TX140

A total of 6 adult sheep were used and 8 osteotomies, 8 mm in diameter and 10 mm deep were formed bilaterally proximal and distal humerus and femur on each animal. Three treatment groups were included in the study, osteoinductive iliac crest bone graft (+Control), empty/no treatment (-Control) and TX140 treated site. The treatment groups were randomly distributed to allow inter- and intra-animal comparison at 6 weeks and 12 weeks timepoints. At different time points, host cell interaction with TX140 and its capability to support bone

healing were assessed and compared with iliac crest (+Control) treated sites. Summary of assessments, the predefined acceptance criteria and the outcomes are presented in Table. S.

H&E staining of the treatment sites after 6 weeks are shown in Figure. S10. TX140 demonstrated granulomatous inflammation mostly comprising foamy macrophages and multinucleate giant cells admixed with small foci of lymphocytes and plasma cells (arrows). Small pools of TX140 could be detected at the treatment sites, 6 weeks post implantation as the degradation rate of the product in bony defects is slower than that in subcutaneous/ dermal models. Sites treated with +Control had inflammatory infiltrates of foamy macrophages admixed with relatively higher numbers of lymphocytes and plasma cells (arrows) and numbers of multinucleate giant cells. Islands of new bone formation (asterisks in Figure. S11) and cellular non-ossified mesenchymal matrix with +Control. The sites treated with autologous graft displayed the highest percentage of new bone rimmed by plump osteoblasts and multinucleated osteoclasts (*i.e.* active bone remodeling).

Table. S8. Bone healing application of TX140. Summary of performed tests, acceptance criteria, acquired results and the associated protocol pass/fail conclusion.

**Test(s) being Performed**	**Acceptance Criteria**	**Results**	**Protocol Pass/Fail**
Product administration	Direct injectability of TX140 into a sheep osteotomy model and adhesion/retention of the product at the site during the operation	TX140 could be successfully injected into the defect site, the product forms hydrogel and adhered despite the presence of active bleeding at the site	**PASS**[1]
Product safety (local interaction)	Equal or less inflammatory response to TX140 compared to +Control (autologous iliac	Hematoxylin and Eosin (H&E)-stained, decalcified sections of lesion explants were used. 6-weeks post operation, TX140 treated sites demonstrate granulomatous inflammation mostly comprising foamy macrophages and multinucleate giant cells admixed with small foci of	**PASS**

	crest group).	lymphocytes and plasma cells (**arrows**). Iliac crest/autologous graft site (+Control) has inflammatory infiltrates of foamy macrophages admixed with relatively higher numbers of lymphocytes and plasma cells (**arrows**).<br>After 12 weeks, TX140 treated sites frequently contain foci of epithelioid macrophages and multinucleate giant cells (**arrows**) admixed with lymphocytes that are centered around large lakes of partially hydrolyzed biomaterial (**asterisks**). +Control sites contain residual inflammatory foci mostly comprising foamy macrophages with lymphocytes associated with foci of partially hydrolyzed biomaterial. +Control sites contain mild inflammatory foci comprising lymphocytes (**arrows**) with occasional foamy macrophages.	
Integration with hard tissue	Comparable or equal bone formation in TX140 treated sites compared to +Control group at 12 weeks post-operation.	After 12 weeks, Masson's Trichrome-stained, decalcified sections of lesion explants were used. TX140 sites demonstrate healing by compact and trabecular woven bone with few residual islands of fibrovascular connective tissue (**asterisk**). G4 sites demonstrate the highest percentage of bone formation interspersed by well-differentiated, paucicellular medullary adipose. Fibrocartilagenous matrix is minimal for all groups (not shown).	**PASS**

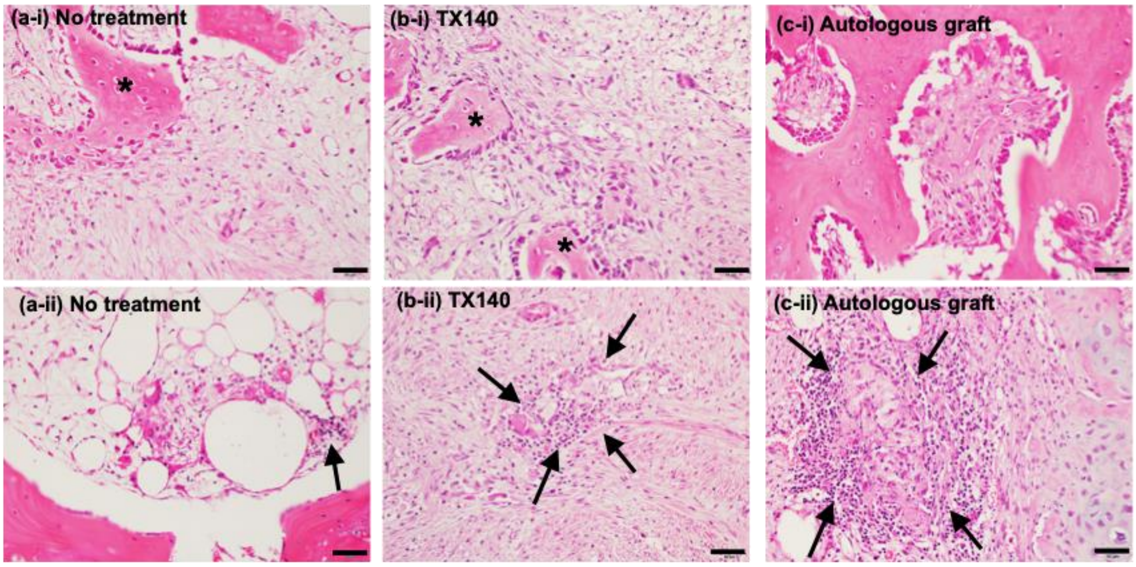

Figure. S10. H&E assessment of bone sites after 6 weeks. 20X magnification photomicrograph composite of Hematoxylin and Eosin (H&E)-stained, decalcified sections of lesion explants from (-Control, no treatment) in a-i and –ii, (TX140) in b-i and b-ii, and (+Control, iliac crest graft) in c-i and –ii. Islands of new bone formation (asterisks) and granulomatous inflammation mostly comprising foamy macrophages and multinucleate giant cells admixed with small foci of lymphocytes and plasma cells (arrows). Scale bars is 50 µm.

H&E staining of the treatment sites after 12 weeks are shown in Figure. S11. TX140 sites contain residual inflammatory foci mostly comprising foamy macrophages with lymphocytes associated with small foci of partially hydrolyzed biomaterial. +Control sites contain mild inflammatory foci comprising lymphocytes (arrows) with occasional foamy macrophages. TX140 sites demonstrate proliferation of anatomizing woven bone trabeculae (WBT) interspersed by mildly inflamed fibrovascular tissue (asterisk) or well-differentiated medullary adipose. +Control (iliac crest autologous graft site) also contain abundant anatomizing woven bone trabeculae (WBT) interspersed by well-differentiated medullary adipose or well-organized fibrous connective tissue (FCT).

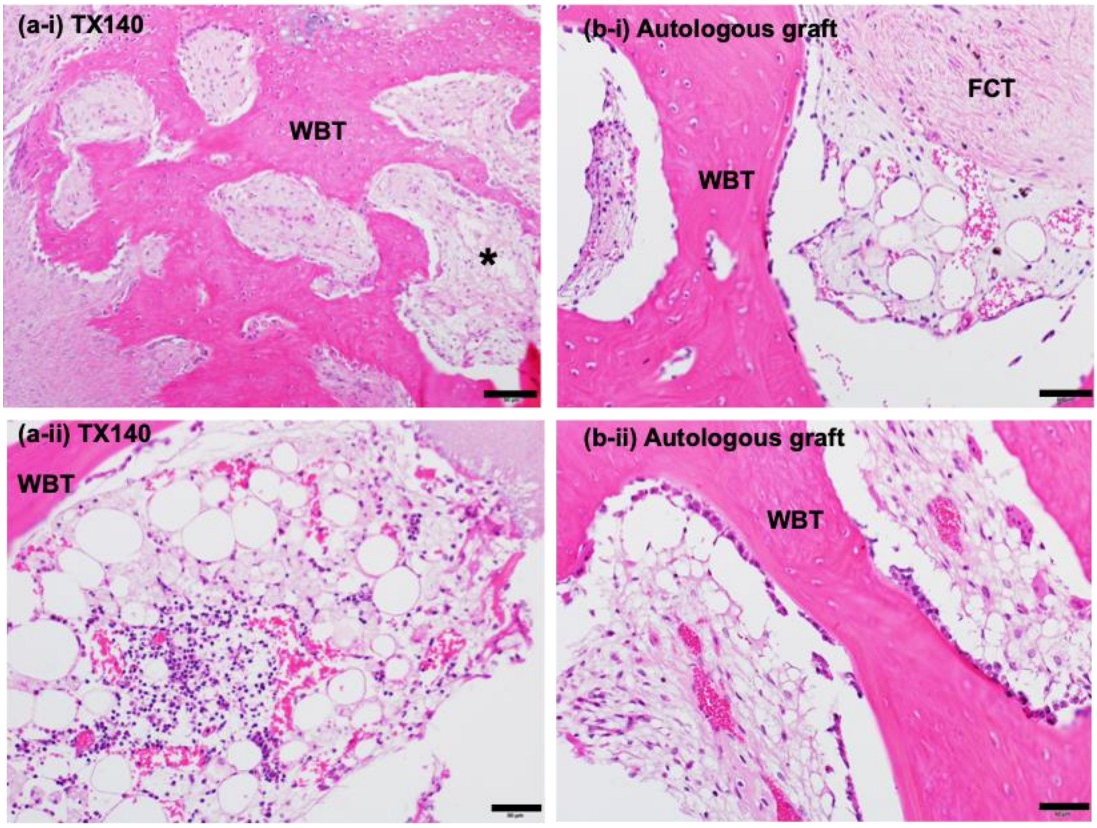

Figure. S11. H&E assessment of bone sites after 12 weeks. 20X magnification photomicrograph composite of Hematoxylin and Eosin (H&E)-stained, decalcified sections of lesion explants. Woven bone trabeculae (WBT), mildly inflamed fibrovascular tissue (asterisk), well-organized fibrous connective tissue (FCT). Scale bars is 50 µm.

Clinical application of TX140 for soft and hard tissue repair

Ten (10) patients were enrolled in this study. All participants were eligible to be a part of this clinical investigation in accordance with the inclusion and exclusion criteria outlined in the clinical investigation plan.

Inclusion criteria involve:

- Patients ≥18 years of age
- Patients undergoing a planned tooth extraction at the centers included in this study
- Patients' willing to give written informed consent and willing to participate in and comply with the study.

Exclusion criteria involves

- Patients with chronic inflammation, out of range blood markers:
    - Erythrocyte sedimentation rate (ESR) above 22 mm for male and 29 mm for female candidates.
    - C-reactive proteins (CRP) above 5 mg/L.
- Patients with low liver function, as indicated by:
    - ALT and/or AST level > 1.5 times the upper limit of normal (ULN).
    - Alkaline phosphatase above 140 IU/L.
    - INR below 0.8 or above 1.3
    - Bilirubin above 1.2 mg/dL.
- Patients with low kidney function, as indicated by:
    - Serum creatinine above 1.4 for both male and female (the norm for male is 1.4 and for female is 1.2).
    - Glomerular filtration rate (GFR) of below 60 ml/min/1.73m^2).
- Patients <18 years of age.
- Pregnant or lactating women, or women of childbearing potential who are not willing to avoid becoming pregnant during the study.
- Patients who are concurrently enrolled in another clinical study or have received an investigational new drug within the past four (4) weeks.
- Patients with a history of a psychological illness or condition such as to interfere with the patient's ability to understand the requirements of the study.
- Patients unwilling or unlikely, in the Principal Investigator's opinion, to comply with the study follow-up.
- Patients with a history of disease(s) that is (are) likely to interfere with the normal post-op healing process or metabolism or excretion of the test medication.

- Patients with an American Society of Anaesthesiologists (ASA) physical status classification of > ASA 2.
- Patients with active infection at the target site or a surgical site located near infection.
- Patients in which the bone surrounding the target site is not viable or is incapable of supporting or anchoring the implant.
- Patients with abnormal calcium metabolic bone disease or immunological abnormalities.
- Patients who are undergoing or are to undergo an immunosuppressive therapy.
- Patients who are undergoing or are to undergo chemotherapy or radiation therapy at or near the implant site.
- Patients with active cancer.

Study participants was followed-up for three (3) months post tooth extraction and treatment with TX140. A summary of patient demographics is presented in Table S9.

Table S9. Clinical trial participants' demographic. Summary of participants' demographic in the clinical investigation.

Number of Participants	N=10
Number of Male Participants	M=10
Number of Female Participants	F=0
Number of participants with smoking history	S=2
Minimum age at the time of operation	Min-age= 28
Maximum age at the time of operation	Max-age=73
Median age of participants	Med-age=51
Average age of participants	Ave-age=53

There was no report of device malfunction throughout the clinical investigation. TX140 was provided in a ready-to-use format; this negates the need for pre-mixing and any

other preparation step by the clinicians prior to the operation. Results in Figure. S12 showed 100% successful rate in the application and gelation of TX140 for all 10 patients. Upon the application of TX140 in all 10 participants, the product form hydrogel within few seconds post injection, it adhered to the site and thus negated the need for primary closure, e.g., membrane or other physical containment.

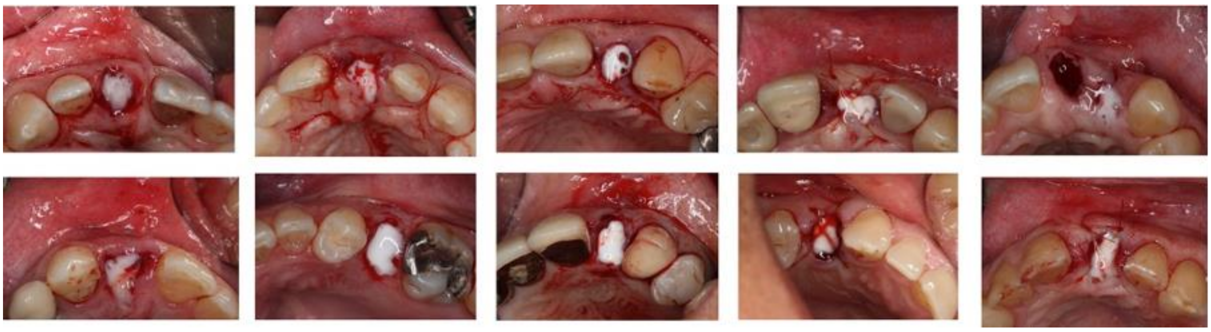

Figure. S12. Sites treated with TX140 in the clinical trial. TX140 application post tooth extraction on 10 patients.

All 10 patients treated with TX140 returned for the first follow-up visit, one-week post operation. There was no report of pain or discomfort from any of the patients. During the oral examination (one-week post administration), there was no sign of infection or inflammation at the site. In addition, wound closure and soft tissue formation were examined by the Principle Investigator. In all ten patients, wound closure was noted and expedited soft tissue formation was detected.

Three months post tooth extraction and treatment with TX140, a CT-scan of the treatment site was collected and thereafter patients underwent implant placement procedure. At this point, biopsies were collected from the TX140 administered site. Samples were histochemically analyzed by an independent laboratory (Sonic Clinical Trials Pty LTD). Table S10 summarizes the findings from the safety point of view of TX140. In all analyzed samples, based on results from CT-scan and histological assessment of the treatment sites, there was no evidence of biological abnormality, necrosis, foreign body type giant cell or foreign body reactions.

Table S10. Safety clinical assessment of TX140. Summary of visual observations and histological assessments of the sites treated with TX140 post tooth extraction.

Patient #	In all follow up visits up-to Three Months post Administration of TX140		Three Months Post Administration of TX140		
			CT-Scans	Histochemical Analyses	
	Device Related Adverse Event	Visual Observation (infection, inflammation or Normal)	Appearance of the site with CT-Scans (Infection, Inflammation or Normal[1])	Synopsis	Foreign Body Reaction
001-001	No	Normal	Normal	No	No
001-002	No	Normal	Normal	No	No
001-003	No	Normal	Normal	No	No
001-004	No	Normal	Normal	No	No
001-005	No	Normal	Normal	No	No
001-006	No	Normal	Normal	No	No
001-007	No	Normal	Not Applicable	Not Applicable	Not Applicable
001-008	No	Normal	Normal	No	No
001-009	No	Normal	Normal	No	No
001-010	No	Normal	Normal	No	No

1: Identification as Normal in the CT-scan results denotes the absence of inflammation, infection, necrosis, hypertrophic bone growth, hypertrophic fibrosis

Histochemical analyses showed the formation of interconnected viable bony trabeculae, a mixture of woven and lamellar bone as well as active osteoblasts and osteoclasts (Table S11). In all patients, active periodontal bone remodeling was noted.

Table S11. Clinical efficacy of TX140 at the socket site. Summary of findings from H&E and Masson's trichrome staining of TX140 tested sites.

Patient #	Macroscopic Findings	Histology Microscopic findings	Diagnosis of the site
001-001	Biopsy fresh tissue. The specimen consists of a core of bone measuring 4mm in length by 2mm in diameter.	Sections show the specimen is composed of interconnected viable bony trabeculae, which are a mixture of woven and lamellar bone, confirmed on polarization and Masson staining. The trabeculae are rimmed by a mixture of osteoclasts and osteoblasts, consistent with ongoing remodeling. There is no evidence of necrosis of a foreign body reaction and no specific pathological abnormality is identified. The appearance is keeping with reactive host bone remodeling.	PERIODONTAL - BONE – Reactive host bone remodeling
001-002	Biopsy fresh tissue. The specimen consists of a core of bone measuring 8mm in length by 2mm in diameter.	Sections show the specimen is composed of interconnected viable bony trabeculae, which are a mixture of woven and lamellar bone, confirmed on polarization and Masson staining. The trabeculae are rimmed by a mixture of osteoclasts and osteoblasts, consistent with ongoing remodeling. There is no evidence of necrosis or a foreign body reaction and no specific pathological abnormality is identified. The appearances are keeping with reactive host bone remodeling.	PERIODONTAL - BONE – Reactive host bone remodeling
001-003	Biopsy fresh tissue. The specimen consists of a	Sections show the specimen is composed of interconnected viable bony trabeculae, which are a mixture of woven and lamellar bone, confirmed on polarization and Masson staining.	PERIODONTAL - BONE – Reactive host bone remodeling

Patient #	Macroscopic Findings	Histology Microscopic findings	Diagnosis of the site
	core of bone measuring 12mm in length by 3mm in diameter.	The trabecular are rimmed by a mixture of osteoclasts and osteoblasts, consistent with ongoing remodeling. There is no evidence of necrosis or a foreign body reaction and no specific pathological abnormality is identified. The appearance is in keeping with reactive host bone remodeling.	
001-004	The specimen consists of a core of bone measuring 4mm in length by 2mm in diameter.	Sections show a fragment of tissue comprising variably sized trabeculae of viable bone which has a sclerotic appearance and is composed of a mixture of woven and lamellar bone. Focal ongoing remodeling with an osteoblastic lining is seen and occasional resorption pits are included. Accompanying bone dust is seen. Small fragments of crushed fibrovascular tissue is included in which a lymphocytic infiltrate is noted however foreign material is not identified and a foreign body type giant cell reaction is not noted. Features to suggest sepsis are not seen. There is no evidence of malignancy.	PERIODNTAL - BONE – Viable Sclerotic bone with ongoing Remodeling, no Foreign Material seen
001-005	Specimen site not - indicated on container. The specimen consists of a core bone measuring	Sections confirms the presence of a core of bone much of which is fragmented and which is composed of sclerotic bone comprising woven and lamellar bone, all of which is viable in nature and in which a degree of ongoing remodeling with osteoblastic and focally osteoclastic activity, manifest as resorption is noted. Accompanying slightly crushed	PERIODNTAL - BONE – Viable Sclerotic bone With Remodeling, no Foreign Material noted

Patient #	Macroscopic Findings	Histology Microscopic findings	Diagnosis of the site
	6mm in length by 2mm in diameter. Embedded whole.	fibrovascular stroma is included which a patchy lymphocytic infiltrate is noted and a rare hemosiderin laden macrophage is present. Foreign material is not seen and a foreign bod giant cell reaction is not identified. Features to suggest sepsis are no evident. There is no evidence of malignancy.	
001-006	Specimen site not - indicated on container. The specimen consists of a core of bone measuring 5mm in length by 2mm in diameter. Embedded whole (1 block) RA12	Sections show a core of bone composed of interconnected trabeculae of viable sclerotic mature lamellar and woven bone is included with intervening loose fibrovascular tissue and bone dust. Evidence of prior remodeling with prominent cement lines with scattered bland osteoblasts are present and the osseous trabeculae are viable throughout. Bone dust is included however convincing foreign material and foreign body type giant cell reaction is not noted. Features to suggest sepsis are not seen. There is no evidence of malignancy.	PERIODNTAL - BONE – Sclerotic Viable bone with evidence of pre-remodeling, no other Abnormality Noted
001-007	Not applicable	Not applicable	Not applicable
001-008	Specimen site not - indicated on container. The specimen consists of a	Sections confirm the presence of a core of bone composed of mature lamellar and somewhat compact bone merging with slightly thickened trabecular in which a combination of both woven and lamellar bone is seen in keeping with a degree of remodeling. The osseous	PERIODNTAL - BONE – Viable bone with ongoing remodeling No other abnormality

Patient #	Macroscopic Findings	Histology Microscopic findings	Diagnosis of the site
	core of bone measuring 5mm in length by 2mm in diameter. Embedded whole. (1 block) RF2	component is viable throughout with osteoblastic and osteoclastic activity is noted. A foreign material is not seen and featured to suggest sepsis are not identified. There is no evidence of malignancy	seen
001-009	Biopsy fresh tissue. The specimen consists of a core of bone measuring 6mm in length by 2mm in diameter.	Sections confirm the presence of a core of bone much of which comprises compact mature lamellar bone of cortical origin merging with interconnected trabeculae of bone with a more cancellous appearance which is moderately thick centrally composed of woven bone but exhibiting peripheral lamination in keeping with maturation. Focally a degree of ongoing remodeling with osteoblastic activity is noted. The companying stroma has a loose fibrovascular appearance. The bone throughout is viable with osteocytes visible within the lacunae. A foreign body type giant cell reaction is not seen. There is no evidence of sepsis. There is no evidence of malignancy.	PERIODNTAL - BONE – Cortical and cancellous bone with evidence of ongoing remodeling Foreign body reaction not noted No sepsis
001-010		Section confirm the presence of a core bone in which an elongated strip of compact lamellar bone is included, bordered at its perimeter by reactive appearing woven bone which itself can be seen to be undergoing lamellation at the perimeter and which mergers with bone with more cancellous architecture but composed of	PERIODNTAL - BONE – Viable woven bone and mature lamellar bone No evidence of foreign material

Patient #	Macroscopic Findings	Histology Microscopic findings	Diagnosis of the site
		interconnected trabecular of largely woven bone within a loose, slightly oedematous and well vascularized stroma with dilated thin walled vascular channel. A degree of bone remodeling within area of osteoclastic and osteoblastic activity are seen. The osseous components is viable throughout, foreign material is not and foreign body type giant cell reaction is not noted. Features suggest sepsis are not identified. The Masson trichrome stain confirms these findings. There is no evidence of malignancy.	No foreign body reaction No evidence of sepsis
